# Macrophages downregulate NEDD9 to counteract *S*. Typhimurium- mediated FAK-AKT activation and lysosome inhibition

**DOI:** 10.1038/s41419-025-07634-9

**Published:** 2025-06-12

**Authors:** Julia Fischer, Lisa Rusyn, Frederike Krus, Liudmila Lobastova, Marc Herb, Alexander Gluschko, Zahra Hejazi, Nina J. Hos, Chiara Calabrese, Jannik Stemler, Petra Mayer, Ruth Hanssen, Sebastian J. Theobald, Jörg Janne Vehreschild, Jonel Trebicka, Martin Krönke, Jochen W. U. Fries, Clara Lehmann, Phuong-Hien Nguyen, Jan Rybniker, Nirmal Robinson, Tamina Seeger-Nukpezah

**Affiliations:** 1 University of Cologne, Faculty of Medicine and University Hospital of Cologne, Department I of Internal Medicine, Center for Integrated Oncology, Cologne, Germany; 2 University of Cologne, Faculty of Medicine and University Hospital of Cologne, Center for Molecular Medicine Cologne (CMMC), Cologne, Germany; 3 German Center for Infection Research (DZIF), Partner Site Bonn-Cologne, Cologne, Germany; 4 University of Münster, Faculty of Medicine and University Hospital of Münster, Department of Internal Medicine B, Albert-Schweitzer-Campus, Münster, Germany; 5 Cologne Excellence Cluster on Cellular Stress Responses in Aging-Associated Diseases (CECAD), Chair Translational Research, Faculty of Medicine and University Hospital Cologne, University of Cologne, Cologne, Germany; 6 University of Cologne, Faculty of Medicine and University Hospital of Cologne, Institute of Medical Microbiology, Immunology and Hygiene, Cologne, Germany; 7 Department of Clinical Research, Clinic of Infectious Diseases, London School of Hygiene and Tropical Medicine, WC1E 7HT, London, United Kingdom; 8 Max Planck Institute for Biology of Ageing, Cologne, Germany; 9 Department II of Internal Medicine, Hematology/Oncology and Infectious Diseases, University Hospital of Frankfurt, Frankfurt, Germany; 10 University of Cologne, Faculty of Medicine and University Hospital of Cologne, Policlinic for Endocrinology, Diabetes and Preventive Medicine, Cologne, Germany; 11 Max Planck Institute for Metabolism Research, Cologne, Germany; 12 European Foundation for the Study of Chronic Liver Failure - EF Clif, Barcelona, Spain; 13 Institute for Bioengineering of Catalonia, Barcelona, Spain; 14 Department of Medical Gastroenterology and Hepatology, University of Southern Denmark, Odense, Denmark; 15 University of Cologne, Faculty of Medicine and University Hospital of Cologne, Institute of Pathology, University of Cologne, Cologne, Germany; 16 Center for Cancer Biology, University of South Australia and SA Pathology, Adelaide, SA Australia; 17 Adelaide Medical School, Faculty of Health and Medical Sciences, The University of Adelaide, Adelaide, SA Australia

**Keywords:** Infection, Infection

## Abstract

The scaffolding protein NEDD9 coordinates signaling downstream of integrins by interacting with focal adhesion kinase (FAK) and thereby promotes cell migration. NEDD9 expression is altered in a number of clinical conditions such as cancer, but its role in innate immunity against infections remains elusive. Transcriptome analysis of *Salmonella* Typhimurium (*S*T)-infected murine macrophages showed downregulation of *NEDD9* and genes belonging to its signaling network. Bacterial infections induced host-mediated lysosomal degradation of NEDD9 in macrophages and PBMCs isolated from patients suffering from bloodstream infection. However, *S*T induced translocation of NEDD9 from the cytoplasm to *S*T-containing phagosomes and prevented their phagolysosome-mediated clearance by FAK/AKT activation, reflecting a bacterial evasion mechanism. Complete loss of NEDD9 significantly reduced bacterial burden and enhanced inflammation upon *S*T infection both in vitro and in vivo. Mechanistically, we show that NEDD9 activates the FAK-AKT pathway allowing phosphorylation of FAK and AKT to impair phagolysosomal-mediated clearance of bacteria. Our study has thus identified NEDD9 as a critical regulator of lysosomal function in macrophages and a potential host-directed therapeutic target to treat bacterial infections.

**Classification:** Biological Sciences, Microbiology

**Figure:**
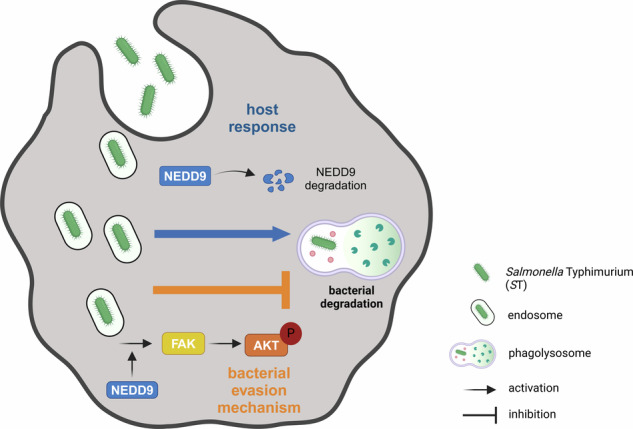
**Macrophages downregulate NEDD9 to counteract** ***S*T mediated FAK-AKT activation**. Upon infection with *S*T NEDD9 is translocated from the cytosol to *S*T-containing phagosomes. Loss of NEDD9 results in enhanced lysosomal capacities supporting bacterial clearance. Strikingly, *S*T recruits and activates FAK and AKT to suppress endosome-lysosome fusion, thereby bypassing lysosome-mediated pathogen clearance. Created in BioRender. Robinson, N. (2021) BioRender.com/n17r483.

**Macrophages downregulate NEDD9 to counteract** ***S*T mediated FAK-AKT activation**. Upon infection with *S*T NEDD9 is translocated from the cytosol to *S*T-containing phagosomes. Loss of NEDD9 results in enhanced lysosomal capacities supporting bacterial clearance. Strikingly, *S*T recruits and activates FAK and AKT to suppress endosome-lysosome fusion, thereby bypassing lysosome-mediated pathogen clearance. Created in BioRender. Robinson, N. (2021) BioRender.com/n17r483.

## Introduction

Bloodstream infections (BSI) caused by bacteria often lead to severe clinical disorders in patients, potentially resulting in life-threating conditions such as sepsis, septic shock, and organ dysfunction [1, 2]. Sepsis remains the cause of approximately six million deaths worldwide, and is frequently underdiagnosed at an early stage when it is still reversible [3]. Moreover, the global emergence of multidrug-resistant pathogens is causing a rise in antibiotic-resistant infections, contributing to high morbidity and mortality of septic patients [4]. Thus, the WHO recently identified management of sepsis as a global health priority in order to improve prevention, diagnosis and clinical management [3]. Better understanding of the molecular mechanisms of BSI to establish alternative therapeutic strategies is essential for better management of bacterially-induced sepsis.

Macrophages are key cells that mediate an effective innate response against pathogens in the bloodstream. Invading pathogens are detected by pattern recognition receptors and degraded within the phagosomes after fusion with lysosomes [5]. Pro-inflammatory responses against pathogens often involve enhanced phagosome processing. Conversely, intracellular pathogens such as *Salmonella* can impair phagolysosomal degradation as a primary strategy to evade degradation by macrophages and reduce production of immunogenic peptides [6–9]. For example, the gastroenteritis causing non-typhoid *Salmonella* (NTS) strain *Salmonella enterica* serovar Typhimurium (*S*. Typhimurium, subsequently referred to as *S*T) actively suppresses phagolysosomal degradation by recruiting focal adhesion kinase (FAK) to *S*T-containing vacuoles [10]. Importantly, *S*T actively invades the bloodstream, particularly in immune-compromised patients, which can cause life-threatening conditions [11]. Recently, the numbers of invasive NTS associated infections have been on the rise, not only in low- and middle- income countries but also in high-income regions such as Australia [12] and Europe [13]. One reason is that treating infections caused by antibiotic-resistant *Salmonella* isolates is increasingly challenging due to a lack of new antibiotics.

NEDD9 (Neural Precursor Cell Expressed, Developmentally Down-Regulated 9) - also known as CAS-L (CRK-associated substrate-related protein) or HEF1 (Human enhancer of filamentation 1) - is a member of the CAS family. The CAS family is a group of non-catalytic scaffolding proteins regulating normal and pathological cellular signaling, thus modifying cell proliferation, adhesion, migration, and differentiation. They are characterized by protein interaction domains, including Src homology 3 (SH3) domain and a substrate domain, which allow them to interact with a wide range of signaling molecules and cytoskeletal components [14]. NEDD9 is frequently overexpressed and predictive for poor clinical outcome in numerous types of tumors [15–17]. Furthermore, NEDD9 enhances immune cell functions including chemokine-induced lymphocyte migration [18–21] and inflammatory responses in rheumatoid arthritis [22]. Upon activation of various cell surface receptors - as best described for integrins - NEDD9 forms complexes with FAK and other kinases to modify intracellular signaling pathways, promoting cell proliferation, migration, cytoskeletal remodeling and adhesion [16, 23, 24]. *Nedd9* knockout mice are both viable and fertile, without a shortened lifespan. Nonetheless, differences in the distribution and quantity of immune cells have been observed in these mice, including an increased number of macrophages in the peripheral blood and spleen [16, 21]. However, whether NEDD9 plays a role in general or specifically during antibacterial host defenses has not yet been investigated.

In this study, we demonstrate a novel, functional role of NEDD9 in macrophage defense against bacterial pathogens. Initially, we identified NEDD9 and known NEDD9 interaction partners being significantly downregulated in murine macrophages upon *S*T infection. We analyzed murine bone-marrow derived macrophages (mBMDMs) from *Nedd9*^*wt/wt*^ and *Nedd9*^*-/-*^ mice as well as human macrophages, and found NEDD9 to be significantly and consistently downregulated and degraded by lysosomes upon infection with various bacteria, including *S*T. Genetic loss of NEDD9 strongly improved both bacterial clearance and secretion of pro-inflammatory cytokines, in vitro and in vivo. Furthermore, *S*T infection induced translocation of NEDD9 to *S*T-containing phagosomes, leading to activation of FAK-AKT. Conversely, loss of NEDD9 inhibited FAK-AKT-activation and enhanced phagolysosomal clearance of *S*T. Overall, our data suggest that downregulation of NEDD9 is a critical host defense mechanism of macrophages against bacterial infections, which is bypassed by *S*T.

## Results

### NEDD9 is downregulated in murine macrophages upon bacterial infection

**Fig. 1:**
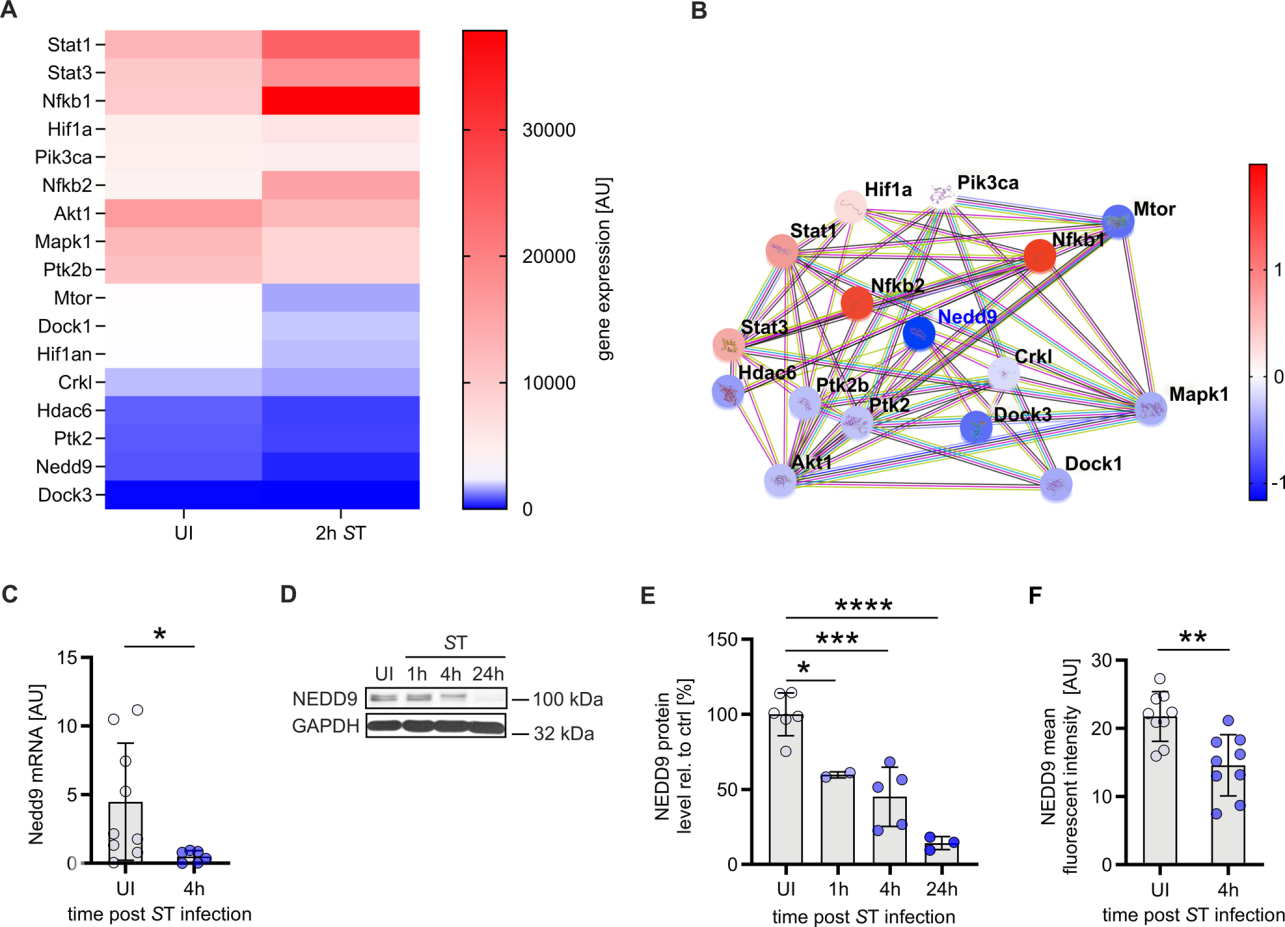
Bacterial infection of murine macrophages leads to transcriptional and post-transcriptional downregulation of NEDD9. **A** Heat map of microarray analysis of murine bone marrow-derived macrophages (mBMDMs) infected with *S*. Typhimurium (*S*T) for 2 hours compared to uninfected control macrophages (UI). Gene expression of UI and infected mBMDMs as arbitrary unit (AU), red for high expression, blue for low expression. *N* = 3, biological replicates. **B** STRING Analysis of functional network of the NEDD9 signaling axis for significantly altered gene expression of the analysis described in (**A**). Genes are highlighted by coloring them according to their fold-change in expression. **C** Real-time PCR analysis of Nedd9 levels normalized to GAPDH by calculation of Δ CT values from mBMDMs infected with *S*T, unpaired t-test: *p* (UI vs. 4 h) = 0.0424, experiment *n* = 3 with at least 3 technical replicates. **D** Western blot analysis of mBMDMs uninfected (UI) and infected with ST at indicated time points followed by (**E**) densitometric analysis of NEDD9 protein level normalized to GAPDH, *n* = 5, biological replicates, one-way ANOVA: *p* (UI vs. 1 h) = 0.0162, *p* (UI vs. 4 h) = 0.0001, *p* (UI vs. 24 h) < 0.0001. **F** Quantification of immunofluorescent staining of intrazellular NEDD9 protein, UI n = 9, *S*T 4 h *n* = 11, biological replicates, unpaired t-test: *p* = 0.0019. A *p*-value > 0.05 equals not significant (ns), *p ≤ 0.05, **p ≤ 0.01, ***p ≤ 0.001, ****p ≤ 0.0001.

To assess the importance of NEDD9 in innate immune responses to bacterial infections, we reanalyzed a previously performed transcriptomics data analysis of wildtype mBMDMs 2 hours post *S*T infection and compared them to uninfected control cells [25]. We found that *Nedd9* mRNA was significantly downregulated (approx. 95%). *Nedd9* mRNA downregulation was accompanied by significant downregulation of known *Nedd9* direct interactors such as *Fak* (also known as *Ptk2*), *Ptk2b* and *Crkl* as well as crucial *Nedd9* signaling partners including *Mapk1* and *Dock1* (Fig. 1A, B) [16, 17, 26, 27]. *Nedd9* downregulation could be confirmed in qRT-PCR from macrophage lysates infected with ST at 4 h timepoint (Fig. 1C). These results suggest a role for NEDD9 in host cell autonomous immune responses towards bacterial infection. In order to further elucidate the role of NEDD9 upon bacterial infection of macrophages, we infected mBMDMs with *S*T and analyzed NEDD9 protein levels by Western Blot analysis and immunofluorescence microscopy at timpoints 0.25, 0.5, 1, 4 and 24 hours. Consistent with our transcriptome and RNA data, we found NEDD9 significantly decreased at 4 hours post *S*T infection (Fig. 1D–F, Supplementary Fig. S1A) and almost completely depleted at 24 hours post infection (Fig. 1D,E).

Induction of macrophage defense mechanisms often depends on the degree of pathogen-mediated virulence [7]. To determine if these factors influenced control of NEDD9 expression, we next analyzed NEDD9 protein levels upon infection of mBMDM with *S*T *Salmonella* Pathogenicity Island SPI1 (Δ*invA*) and SPI2 (Δ*ssaV*) mutants, which have defects in the main type III secretion system (T3SS). NEDD9 levels were diminished despite the lack of SPI1 and SPI2 genes, suggesting that the decline in NEDD9 was independent of *S*T-specific virulence factors (Supplementary Fig. S1B**)**. Next, we investigated if decreased NEDD9 is specific to *S*T. Therefore, we treated macrophages with ST derived LPS, which is a TLR-4 agonist as well as with Pam3CSK activating TLR-2. Interestingly, LPS alone was sufficient to completely decrease NEDD9 levels (Supplementary Fig. S1C) indicating that Nedd9 downregulation is specific for Gram-negative pathogens. This finding was further supported by western blot analysis of NEDD9 in response to *Staphylococcus aureus* (*SA*) and *Shigella flexneri* (*SF*) infection showing reduced NEDD9 levels only for the Gram-negative pathogen *SF* (Supplementary Fig. S1D–F).

Taken together, these data indicate that *S*T infection dynamically downregulates NEDD9 at both the mRNA and protein levels in a virulence independent manner.

### NEDD9 translocates from cytoplasm to the phagolysosomal complex and inhibits lysosomal degradation upon *S*T infection

To understand the mechanism of NEDD9 downregulation in macrophages during bacterial infection, we analyzed whether the depletion of NEDD9 protein occurs by proteasomal degradation, as previously reported for other stimuli [28, 29]. To this end, we infected mBMDMs with *S*T and treated them with the proteasome inhibitor MG132 followed by Western blotting. However, proteasome inhibition did not prevent NEDD9 degradation (Supplementary Fig. S2A**)**, implying a different degradation mechanism upon infection.

NEDD9 activity is often determined by relocalization from the cytosol to sites of defined cellular processes such as the mitotic spindle during mitosis [30–32] or the basal body in the process of ciliary disassembly [33]. In addition, proteins can also be degraded via the lysosomal pathway. Macrophages mainly eliminate invading pathogens by activating the phagolysosomal machinery. In our previous studies we have shown that host proteins such as SIRT-1 and AMPK traffic into lysosomes for degradation during host-pathogen interactions [7].

**Fig. 2:**
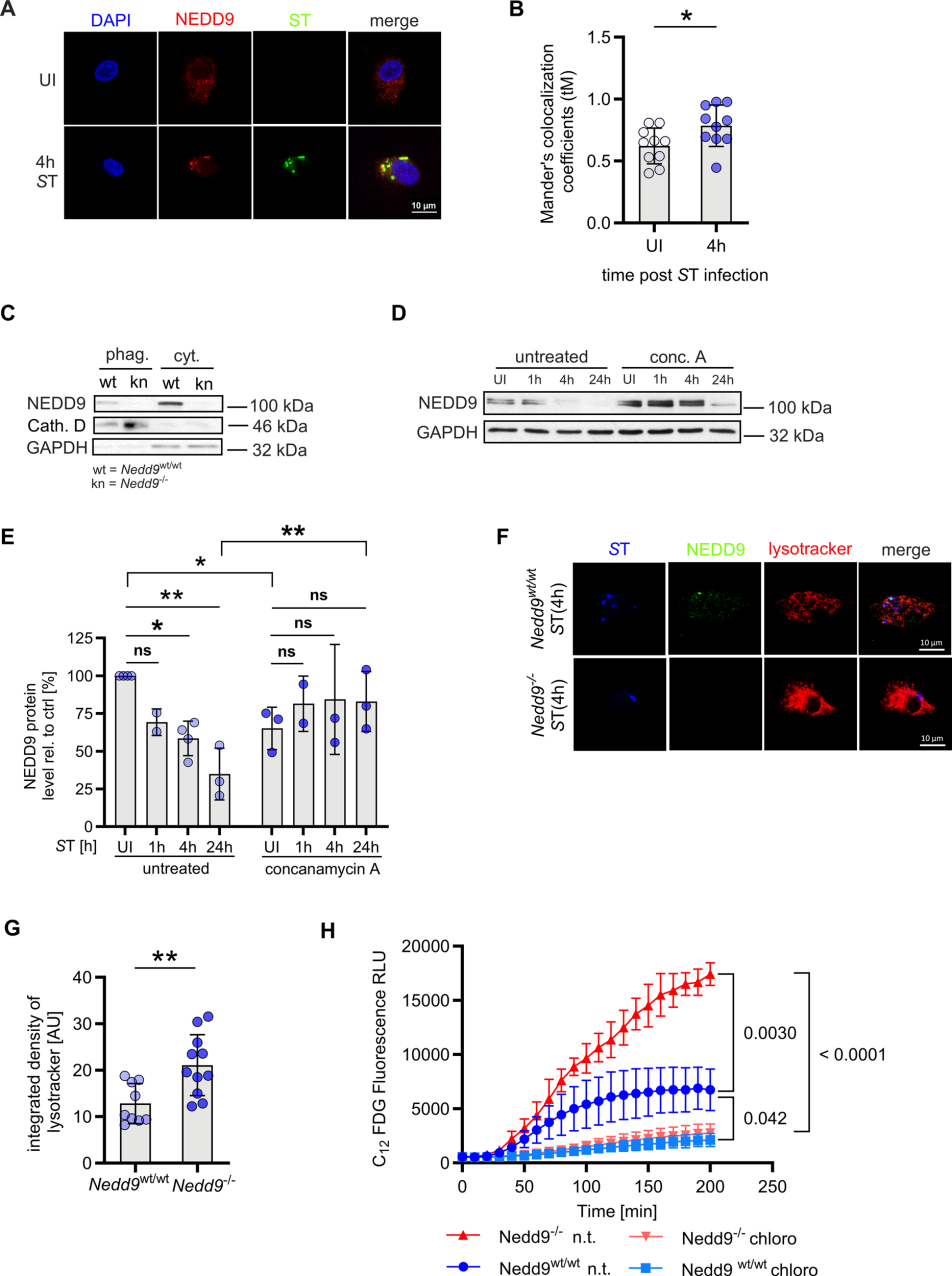
NEDD9 translocates to the phagolysosomal complex and inhibits lysosomal degradation in *S*T infected macrophages. **A** Representative confocal microscopy images of human macrophages uninfected (UI) and infected with *S*T for 4 hours and stained for *S*T (green) and NEDD9 (red). **B** Analysis of colocalization of NEDD9 with LAMP1 using Mander’s colocalization coefficient, *n* = 10, biological replicates, unpaired t-test: *p* = 0.0323. **C** Western blots of isolated phagosomes from *Nedd9*^wt/wt^ and *Nedd9*^-/-^ mBMDMs 4 h post *S*T infection and blotted for NEDD9, phagolysosomal marker Cathepsin D (Cath. D) and cytosolic marker GAPDH, *n* = 2, biological replicates. **D** Infection of mBMDMs with *S*T and parallel treatment with 100 nM concanamycin A (conc. A) followed by Western blotting and (**E**) densitometric analysis of NEDD9 protein level normalized to GAPDH and compared to uninfected cells (UI), *n* = 3, biological replicates, 2-way ANOVA: *p* (untreated UI vs. 4 h) = 0.0235, *p* (untreated UI vs. 24 h) = 0.0012, *p* (untreated UI vs. concanamycin A UI) = 0.0227, *p* (untreated 24 h vs. concanamycin A 24 h) = 0.0049. **F** Representative confocal microscopy images of *Nedd9*^wt/wt^ and *Nedd9*^-/-^ mBMDMs infected with *S*T for 4 hours and stained for *S*T (blue), NEDD9 (green) and lysotracker (red) and (**G**) integrated density analysis of LysoTracker. *Nedd9*^wt/wt^ *n* = 8, *Nedd9*^-/-^ *n* = 11, biological replicates, unpaired t-test: *p* = 0.0045. **H** Measurement of ß-Galactosidase activity by release of C12FDG in *S*T infected *Nedd9*^wt/wt^ and *Nedd9*^-/-^ mBMDMs treated with chloroquine (chloro) or non-treated (n.t.), *n* = 4, biological replicates, 2-way ANOVA: *p* (*Nedd9*^wt/wt^ and *Nedd9*^-/-^ *S*T 200 min.) = 0.0030, *p* (*Nedd9*^-/-^ UT vs. Chloroquin *S*T 200 min.) < 0.0001, *p* (*Nedd9*^wt/wt^ UT vs. Chloroquin *S*T 200 min.) = 0.042. *p ≤ 0.05, **p ≤ 0.01.

To assess whether NEDD9 subcellular localization is altered upon infection, we performed immunofluorescence staining of *S*T infected *Nedd9*^*wt/wt*^ and *Nedd9*^*-/-*^ mBMDMs as well as human macrophages derived from PBMCs of healthy donors. Untreated macrophages showed diffuse cytoplasmic expression of NEDD9. In contrast, NEDD9 clustered and colocalized with *S*T at 4 hours post infection (Fig. 2A) and with lysosomal-associated membrane protein 1 (LAMP-1) (Fig. 2B, Supplementary Fig. S2B, C). This suggests that *S*T infection triggers NEDD9 trafficking from the cytoplasm to the phagolysosome. Consistently, we detected NEDD9 in lysates of isolated *S*T-containing phagosomes 4 h post infection of *Nedd9*^*wt/wt*^ mBMDMs (Fig. 2C). Notably, treatment with vacuolar ATPase inhibitor Concanamycin A significantly stabilized NEDD9 levels upon *S*T infection in mBMDMs (Fig. 2D, E). Interestingly, immunofluorescence staining using LysoTracker to track acidic organelles within macrophages upon *S*T infection revealed, besides colocalization with NEDD9 (Fig. 2A**)** a significantly increased intensity of LysoTracker in *Nedd9*^*-/-*^ mBMDMs compared to *Nedd9*^*wt/wt*^ mBMDMs indicating elevated lysosomal activity in the absence of NEDD9 (Fig. 2F, G**)**. This data was further supported by a significant increase in lysosomal ß-galactosidase activity measured on C_12_FDG coated *S*T indicative of enhanced phagolysosomal processing **(**Fig. 2H**)**. Collectively, these data suggest that in macrophages NEDD9 is degraded by the lysosomal machinery upon *S*T infection. Furthermore, it indicates that downregulation of NEDD9 facilitates phagolysosomal activity in macrophages upon bacterial infection.

### *Nedd9* depletion enhances the inflammatory response of bacteria-infected macrophages and improves their bacterial clearance ex vivo

**Fig. 3:**
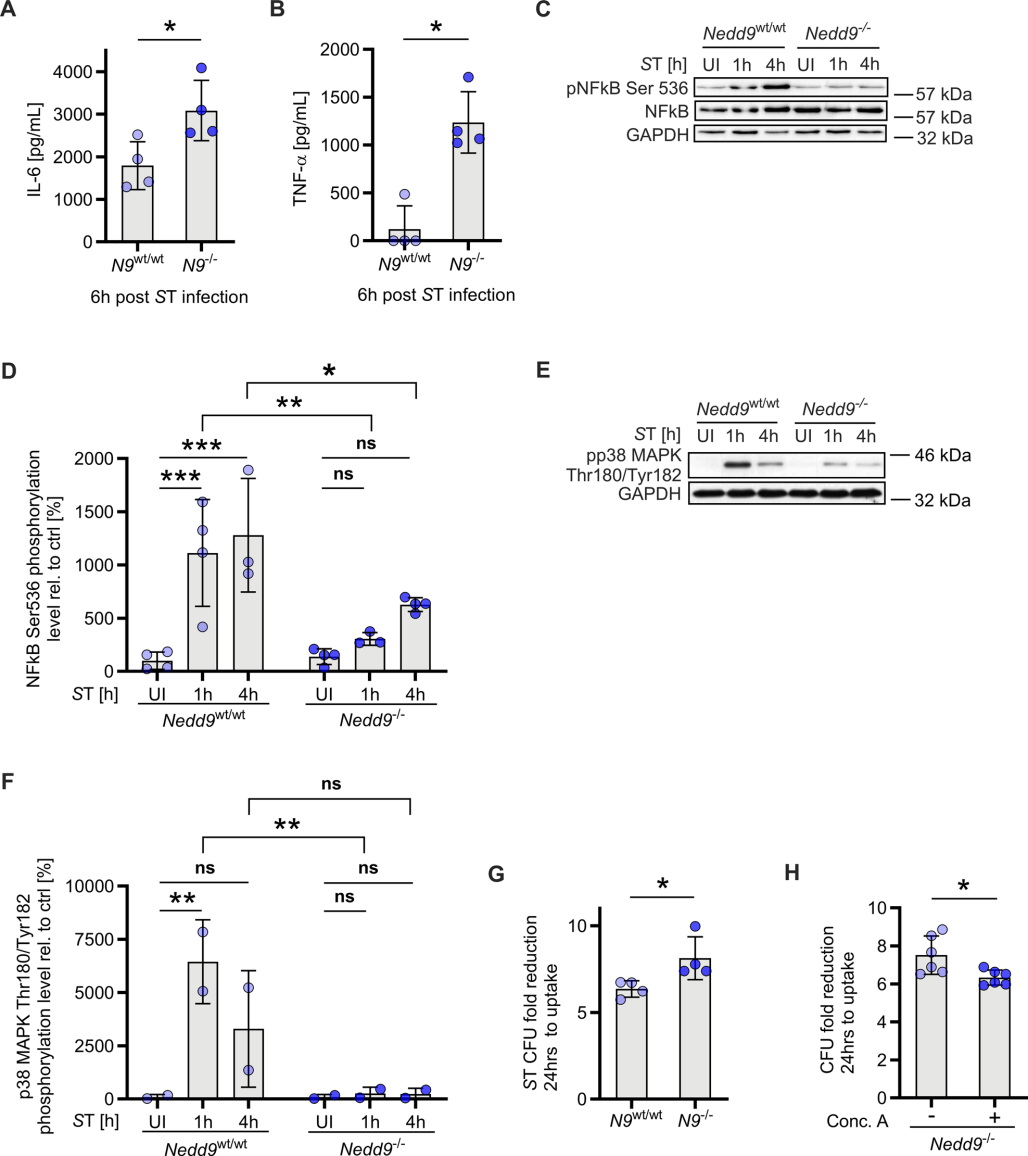
*Nedd9* depletion enhances the pro-inflammatory response of bacteria-infected macrophages and improves their bacterial clearance ex vivo. Infection of *Nedd9*^wt/wt^ and *Nedd9*^-/-^ mBMDMs with *S*T. **A** IL-6, unpaired t-test: *p* = 0.0289 and (**B**) TNF-α, Mann-Whitney test: *p* = 0.0286 in supernatants of mBMDMs 6 h post *S*T infection, *n* = 4, biological replicates. **C** Western blot analysis of NFК-B Ser536 phosphorylation 1 and 4 hours post infection with *S*T and (**D**) densitometric analysis of pNF-КB Ser536 normalized to GAPDH, *n* = 4, biological replicates, 2-way ANOVA: p (*Nedd9*^wt/wt^ UI vs. *S*T 1 h) = 0.0005, *p* (*Nedd9*^wt/wt^ UI vs. *S*T 4 h) = 0.0002, *p* (*Nedd9*^wt/wt^ *S*T 1 h vs. *Nedd9*^-/-^ *S*T 1 h) = 0.0024, *p* (*Nedd9*^wt/wt^ *S*T 4 h vs. *Nedd9*^-/-^ *S*T 4 h) = 0.0103. **E** Western blot analysis of phosphorylated p38 MAPK Tyr180/Tyr182 1 h and 4 h post infection with *S*T and (**F**) densitometric analysis of phosphorylated p38 MAPK Tyr180/Tyr182 normalized to GAPDH, *n* = 2, biological replicates, 2-way ANOVA: *p* (*Nedd9*^wt/wt^ UI vs. *S*T 1 h) = 0.0090, *p* (*Nedd9*^wt/wt^ *S*T 1 h vs. *Nedd9*^-/-^ *S*T 1 h) = 0.0043. **G** In vitro bacterial burden (CFU) in *Nedd9*^wt/wt^ vs. *Nedd9*^-/-^ mBMDMs plotted as fold reduction of 24 h to uptake (0 h), *n* = 3, experiments with at least 3 replicates, Mann-Whitney test: *p* = 0.0286. **H** CFU of *Nedd9*^-/-^ mBMDMs treated with 100 nM Concanamycin A (Conc. A) and controls plotted as fold reduction of 24 h to uptake (0 h), *n* = 2, experiments with 3 replicates each, unpaired t-test: *p* = 0.0235. p> 0.05 equals not significant (ns), *p ≤ 0.05, **p ≤ 0.01, ***p ≤ 0.001.

Next, we investigated how loss of *Nedd9* regulates macrophage defense mechanisms upon *S*T infection. For this purpose, mBMDMs isolated from *Nedd9*^*wt/wt*^ and *Nedd9*^*-/-*^ mice were infected with *S*T and their inflammatory response was quantified by ELISA. Here, we found significantly higher expression of the pro-inflammatory cytokines IL-6 and TNF-α in response to *S*T infection in cell culture supernatants of *Nedd9*-deficient mBMDMs measured by ELISA (Fig. 3A, B). Interestingly, *Nedd9*^*-/-*^ macrophages showed decreased NF-κB and p38 phosphorylation in Western blot analysis suggesting that NF-κb and p38 are regulated by other mechanisms, which are independent of the enhanced pro-inflammatory responses upon loss of *Nedd9* (Fig. 3C–F). Strikingly, the enhanced pro-inflammatory response in *Nedd9*^*-/-*^ macrophages was accompanied by significantly reduced bacterial burden of *S*T in *Nedd9*^*-/-*^ compared to *Nedd9*^*wt/wt*^ mBMDMs, shown as a higher fold reduction of bacterial burden, implying that loss of *Nedd9* enhances the clearance of bacterial infection (Fig. 3G). Furthermore, reduced *S*T burden in *Nedd9*^*-/-*^ mBMDMs could be rescued by lysosomal inhibition using concanamycin A (Fig. 3H). Taken together, our data suggest that NEDD9 downregulation during bacterial infection represents a cell-autonomous mechanism that enhances the antibacterial defenses against *S*T.

### *Nedd9* knockout boosts inflammatory response upon *S*T infection and facilitates bacterial clearance in vivo

**Fig. 4:**
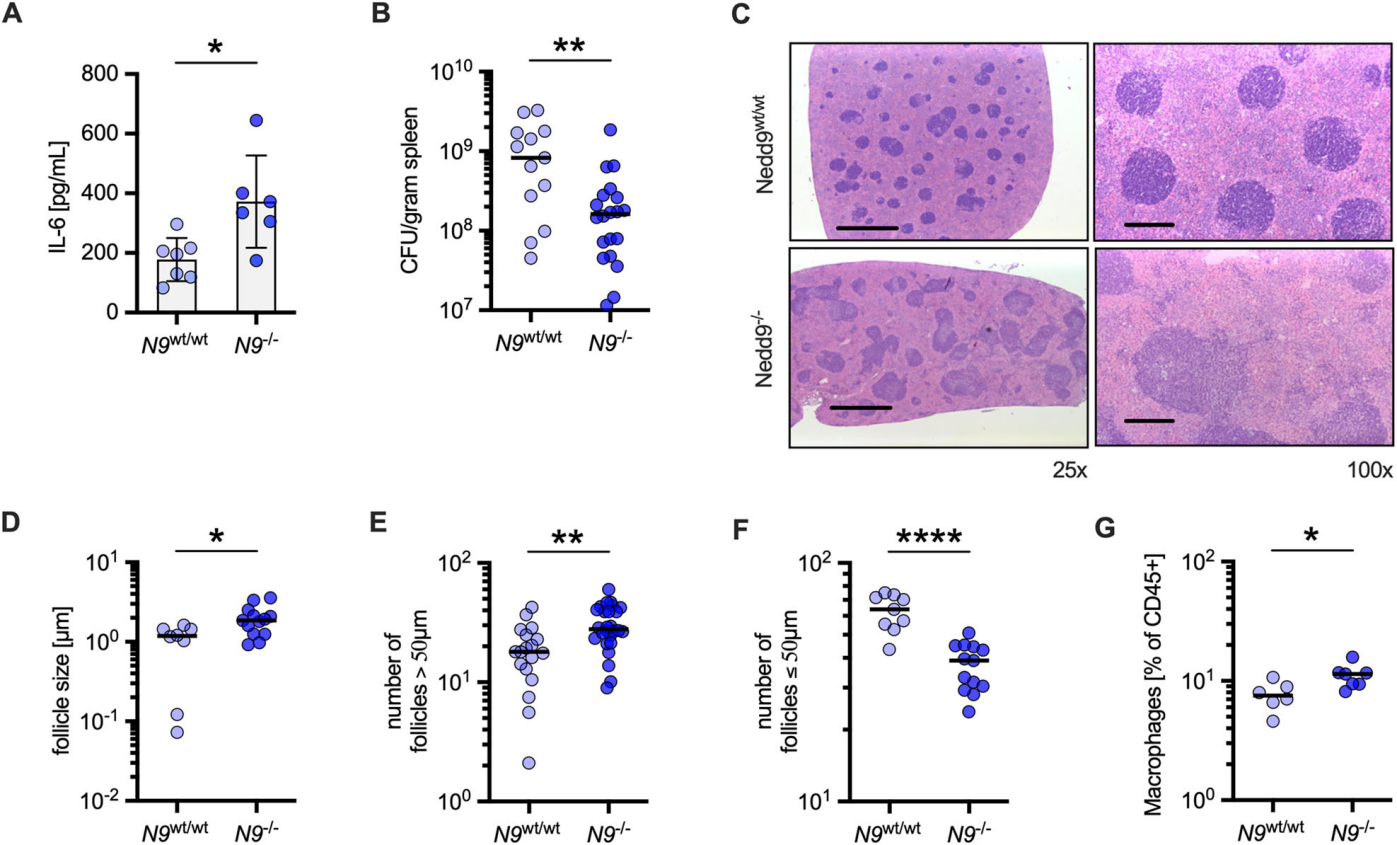
*Nedd9* knockout boosts inflammatory response in *S*T infection and facilitates bacterial clearance in vivo. Infection of *Nedd9*^wt/wt^ and *Nedd9*^-/-^ mice with *S*T for 4 days. **A** IL-6 levels in spleens, *n* = 6 animals/group, *Nedd9*^wt/wt^ vs. *Nedd9*^-/-^, unpaired t-test: *p* = 0.0127. **B** In vivo bacterial burden expressed as colony forming units (CFU) in spleens of *Nedd9*^wt/wt^ and *Nedd9*^-/-^ mice per gram of spleen tissue, *Nedd9*^wt/wt^ *n* = 13, *Nedd9*^-/-^ *n* = 20, Mann-Whitney test: *p* = 0.0072. **C** Representative HE-stained sections of spleens. Scale bars represent 1000 µm at 25x magnification and 200 µm at 100x magnification. **D** Analysis of mean follicle size per spleen area (µm), *Nedd9*^wt/wt^ *n* = 8, *Nedd9*^-/-^ *n* = 13, unpaired t-test: *p* = 0.0119. **E** Number of follicles > 50 µm, *Nedd9*^wt/wt^ *n* = 18, *Nedd9*^-/-^ *n* = 26, unpaired *t*-test: *p* = 0.0021. **F** Number of follicles ≤ 50 µm, *Nedd9*^wt/wt^ *n* = 9, *Nedd9*^-/-^ *n* = 13, unpaired t-test: *p* < 0.0001. **G** Quantification of infiltrating CD11b+ cells (macrophages) as % of CD45+ cells in spleen tissue by flow cytometry, *Nedd9*^wt/wt^ *n* = 6, *Nedd9*^-/-^ *n* = 7, unpaired t-test: *p* = 0.0222. *p ≤ 0.05, **p ≤ 0.01, ****p ≤ 0.0001.

Given the elevated pro-inflammatory response and reduced bacterial load in *Nedd9*^*-/-*^ mBMDMs in vitro, we investigated the impact of *Nedd9* loss on bacterial clearance in vivo by challenging *Nedd9*^*wt/wt*^ and *Nedd9*^*-/-*^ mice intraperitoneally with *S*T. In line with the in vitro data, *Nedd9* loss significantly enhanced the pro-inflammatory responses upon *S*T infection, as demonstrated by elevated cytokine IL-6 in spleens of *S*T-infected *Nedd9*^*-/-*^ compared to *Nedd9*^*wt/wt*^ mice (Fig. 4A**)** while loss of *Nedd9* did not affect body or spleen weight upon infection (Supplementary Fig. S3A, B). In concordance with our in vitro data, *Nedd9* loss significantly reduced *S*T burden in the spleens of mice compared to wildtype animals 4 days post infection (Fig. 4B). In line with a stronger pro-inflammatory response and lower *S*T burden, histopathological analysis of *Nedd9*^*-/-*^ spleens revealed greater infiltration of lymphoid and myeloid cells as evidenced by follicles of significantly larger size and less defined, confluent shape and characterized by immune cells that spread more extensively (Fig. 4C–F). Consistently, flow cytometric analysis of spleens from *Nedd9*^*-/-*^ compared to *Nedd9*^*wt/wt*^ mice indicated significantly enhanced recruitment of CD11b^+^ myeloid cells, including macrophages (Fig. 4G**)**. Thus, these results provide first evidence that NEDD9 downregulation is an important host innate immune defense against bacterial infection both in vitro and in vivo.

### NEDD9 promotes *S*T-mediated FAK-AKT activation and helps *S*T evade lysosomal degradation

**Fig. 5:**
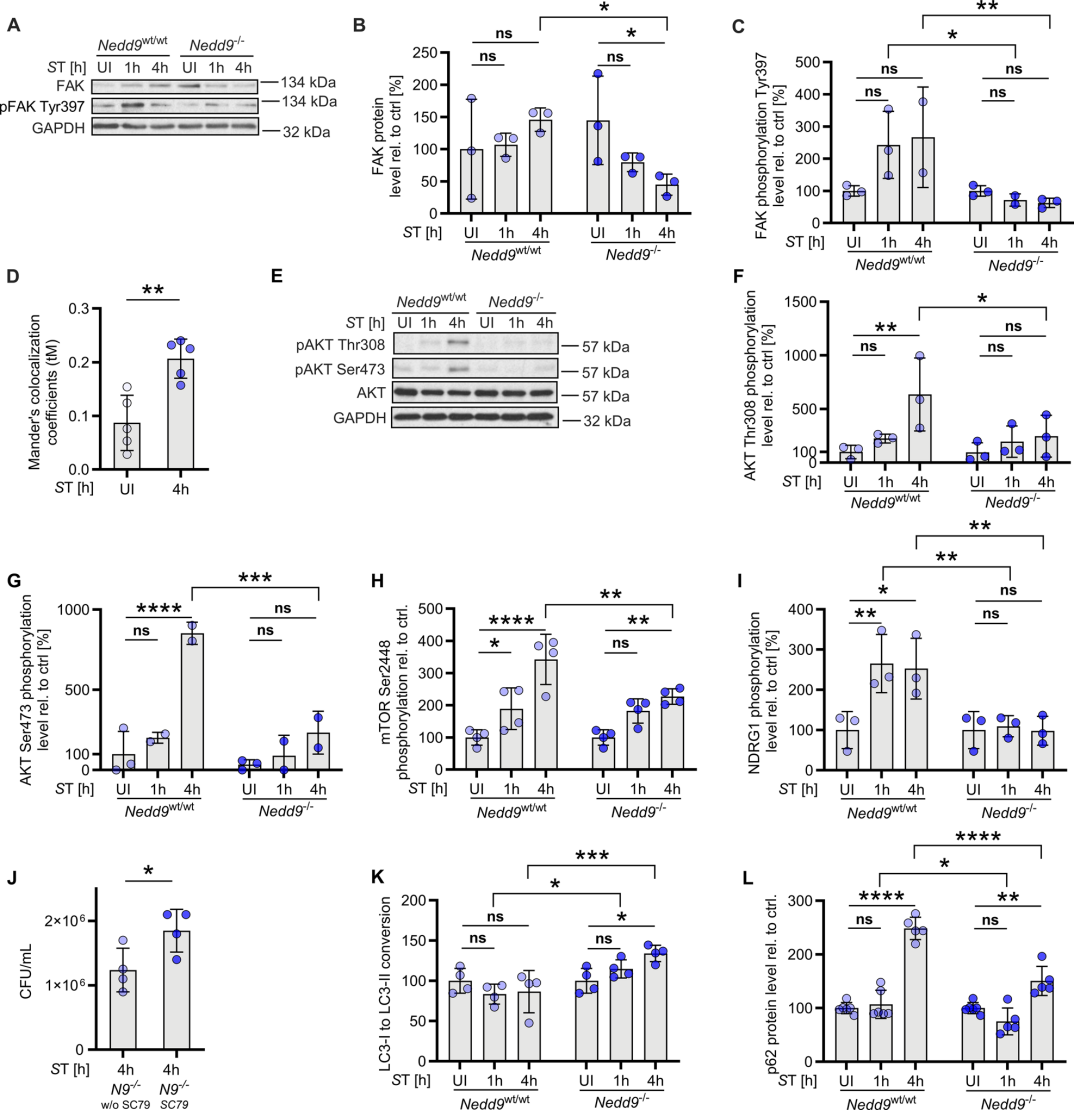
NEDD9 promotes *S*T-mediated FAK-AKT activation and helps *S*T evade lysosomal degradation. **A** Western blot analysis of *Nedd9*^wt/wt^ and *Nedd9*^-/-^ mBMDMs infected with *S*T and (**B**) densitometric analysis of FAK protein level normalized to GAPDH, *n* = 3, 2-way ANOVA: *p* (*Nedd9*^-/-^ UI vs. *Nedd9*^-/-^ *S*T 4 h) = 0.0430, *p* (*Nedd9*^wt/wt^ *S*T 4 h vs. *Nedd9*^-/-^ *S*T 4 h) = 0.0166 and (**C**) phosphorylated FAK protein level normalized to GAPDH 1 and 4 h post *S*T infection, n = 3, 2-way ANOVA: *p* (*Nedd9*^wt/wt^ *S*T 1 h vs. *Nedd9*^-/-^ *S*T 1 h) = 0.0219, *p* (*Nedd9*^wt/wt^ *S*T 4 h vs. *Nedd9*^-/-^ *S*T 4 h) = 0.0090. **D** Infection of *Nedd9*^wt/wt^ and *Nedd9*^-/-^ mBMDMs with *S*T (1 h, 4 h, 24 h post infection, uninfected) and quantification of pFAK and NEDD9 colocalization, *n* = 5, biological replicates, unpaired t-test: *p* = 0.0028. **E** Infection of *Nedd9*^wt/wt^ and *Nedd9*^-/-^ mBMDMs with *S*T and Western blot analysis followed by densitometric analysis, *n* = 3, of (**F**) phosphorylated AKT Thr308 normalized to GAPDH, 2-way ANOVA: *p* (*Nedd9*^wt/wt^ UI vs. *S*T 4 h) = 0.0079, *p* (*Nedd9*^wt/wt^ *S*T 4 h vs. *Nedd9*^-/-^ *S*T 4 h) = 0.0199 and (**G**) phosphorylated AKT Ser473 normalized to GAPDH at indicated time points, *n* = 3, 2-way ANOVA: *p* (*Nedd9*^wt/wt^ UI vs. *S*T 4 h) < 0.0001, *p* (*Nedd9*^wt/wt^ *S*T 4 h vs. *Nedd9*^-/-^ *S*T 4 h) = 0.0003. Densitometric analysis of Western blots showing (**H**) phosphorylated mTOR Ser2448, *n* = 3, 2-way ANOVA: p (*Nedd9*^wt/wt^ UI vs. *S*T 1 h) = 0.0401, *p* (*Nedd9*^wt/wt^ UI vs. *S*T 4 h) < 0.0001, *p* (*Nedd9*^-/-^ UI vs. *S*T 4 h) = 0.0036, *p* (*Nedd9*^wt/wt^ *S*T 4 h vs. *Nedd9*^-/-^ *S*T 4 h) = 0.0029 and (**I**) phosphorylated NDRG1 normalized to GAPDH in *Nedd9*^wt/wt^ and *Nedd9*^-/-^ mBMDMs 1 and 4 h post *S*T infection, 2-way ANOVA: *p* (*Nedd9*^wt/wt^ UI vs. *S*T 1 h) = 0.0067, *p* (*Nedd9*^wt/wt^ UI vs. *S*T 4 h) = 0.0112, *p* (*Nedd9*^wt/wt^ *S*T 1 h vs. *Nedd9*^-/-^ *S*T 1 h) = 0.0038, *p* (*Nedd9*^wt/wt^ *S*T 4 h vs. *Nedd9*^-/-^ *S*T 4 h) = 0.0040. **J** Bacterial burden (CFU/mL) of *S*T infected *Nedd9*^-/-^ mBMDMs untreated and treated for 4 h with 1 µM AKT activator SC-79 in vitro, *n* = 4, unpaired t-test: *p* = 0.0418. Densitometric analysis of Western blots showing (**K**) LC3-I to LC3-II conversion normalized to ß-Actin in *Nedd9*^wt/wt^ and *Nedd9*^-/-^ mBMDMs 1 h and 4 h post *S*T infection, *n* = 3, 2-way ANOVA: *p* (*Nedd9*^-/-^ UI vs. *Nedd9*^-/-^ *S*T 4 h) = 0.0203, *p* (*Nedd9*^wt/wt^ *S*T 1 h vs. *Nedd9*^-/-^ *S*T 1 h) = 0.0128, *p* (*Nedd9*^wt/wt^ *S*T 4 h vs. *Nedd9*^-/-^ *S*T 4 h) = 0.0006 and (**L**) p62, *n* = 3, 2-way ANOVA: *p* (*Nedd9*^wt/wt^ UI vs. *S*T 4 h) < 0.0001, *p* (*Nedd9*^-/-^ UI vs. *S*T 4 h) = 0.0012, *p* (*Nedd9*^wt/wt^ *S*T 1 h vs. *Nedd9*^-/-^ *S*T 1 h) = 0.0179, *p* (*Nedd9*^wt/wt^ *S*T 4 h vs. *Nedd9*^-/-^ *S*T 4 h) < 0.0001. *p* > 0.05 equals not significant (ns), *p ≤ 0.05, **p ≤ 0.01, ***p ≤ 0.001, ****p ≤ 0.0001.

A well described adaptation of *S*T to evade host defense mechanisms is to trigger FAK signaling by recruiting it to and activating it at the surface of *S*T-containing vacuoles [10]. Subsequently, FAK activates AKT, which inhibits autophagy and protects *S*T from lysosomal degradation [10]. We and others have previously shown that inhibition of the FAK effector kinase AKT increases lysosomal functions, resulting in enhanced ability of macrophages to eliminate bacterial pathogens [7, 8, 10]. NEDD9 is a direct interaction partner and substrate of FAK, being recruited by FAK to focal adhesions to regulate cellular adhesion and migration [34, 35]. Given that *S*T infection causes NEDD9 to accumulate at the site of *S*T-containing vacuoles and that NEDD9 expression aids *S*T in evading macrophage-mediated killing, we investigated whether NEDD9 plays a role in this adaptive mechanism of *S*T to escape host defenses. Therefore, we performed immunoblot analysis of *Nedd9*^*wt/wt*^ and *Nedd9*^*-/-*^ macrophages 1 hour and 4 hours post *S*T infection. While NEDD9 wildtype macrophages showed a tendency towards increased levels of total FAK and phosphorylated FAK Tyr397 (pFAK) after *S*T infection, loss of NEDD9 resulted in significantly decreased FAK and pFAK levels upon infection compared to wildtype controls (Fig. 5A–C). In line with our results and NEDD9 being well known to mediate FAK signaling, pFAK markedly colocalized with NEDD9 upon *S*T infection (Fig. 5D, Supplemental Fig. 4A). As expected according to the work of Owens et al. *S*T infection induced AKT phosphorylation at Thr308 and Ser473 in *Nedd9*^*wt/wt*^ macrophages slightly after 1 hour and prominently after 4 hours (Fig. 5E–G). In contrast, NEDD9 loss prevented this sharp increase in AKT phosphorylation in macrophages, particularly at 4 hours post *S*T infection, and resulted in significantly less AKT activation compared to wildtype controls (Fig. 5E–G). Accordingly, phosphorylation of AKT substrates were decreased in *S*T-infected *Nedd9*^*-/-*^ macrophages compared to wildtype controls, with significant changes in phosphorylated mTOR (pMTOR) as well as NDRG-1 (pNDRG-1) and a trend towards reduced expression of S6 kinase (pS6K) (Fig. 5 H, I, Supplemental Fig. 4B–D). Taken together, this indicates that NEDD9 is required for *S*T-induced FAK-AKT signaling. Consistent with this finding, treatment of *Nedd9*^*-/-*^ mBMDMs with the AKT activator SC-79 significantly enhanced *S*T burden at 4 hours (Fig. 5J, Supplemental Fig. 4E) again indicating that downregulation of the FAK-AKT axis enhances lysosomal functions. Since FAK and AKT signaling have been previously shown to regulate autophagy, we analyzed the conversion of LC-3 I-to II, a specific marker of autophagy, which we found to be significantly increased in *Nedd9*^*-/-*^ macrophages upon infection compared to wildtype controls (Fig. 5K, Supplemental Fig. 4F). Further, we analyzed p62 levels, a known receptor protein for the cargo destined for autophagic degradation in *Nedd9*^*wt/wt*^ and *Nedd9*^*-/-*^ macrophages. Here, we observed decreased levels of p62 in *Nedd9*^*-/-*^ macrophages indicating enhanced lysosome-dependent autophagic flux upon infection (Fig. 5L, Supplemental Fig. 4F). This data is consistent with enhanced lysosomal activity observed by increasing cleavage of C12 FDG **(**Fig. 2H**)**. Collectively, our data show that NEDD9 is necessary for functional *S*T-mediated FAK-AKT signaling and subsequent reduction in lysosomal function. Thus, NEDD9 downregulation is a plausible mechanism of the macrophage to prevent the host evasion mechanism of *S*T.

### NEDD9 is downregulated in human macrophages and patients with bacterial bloodstream infections

**Fig. 6:**
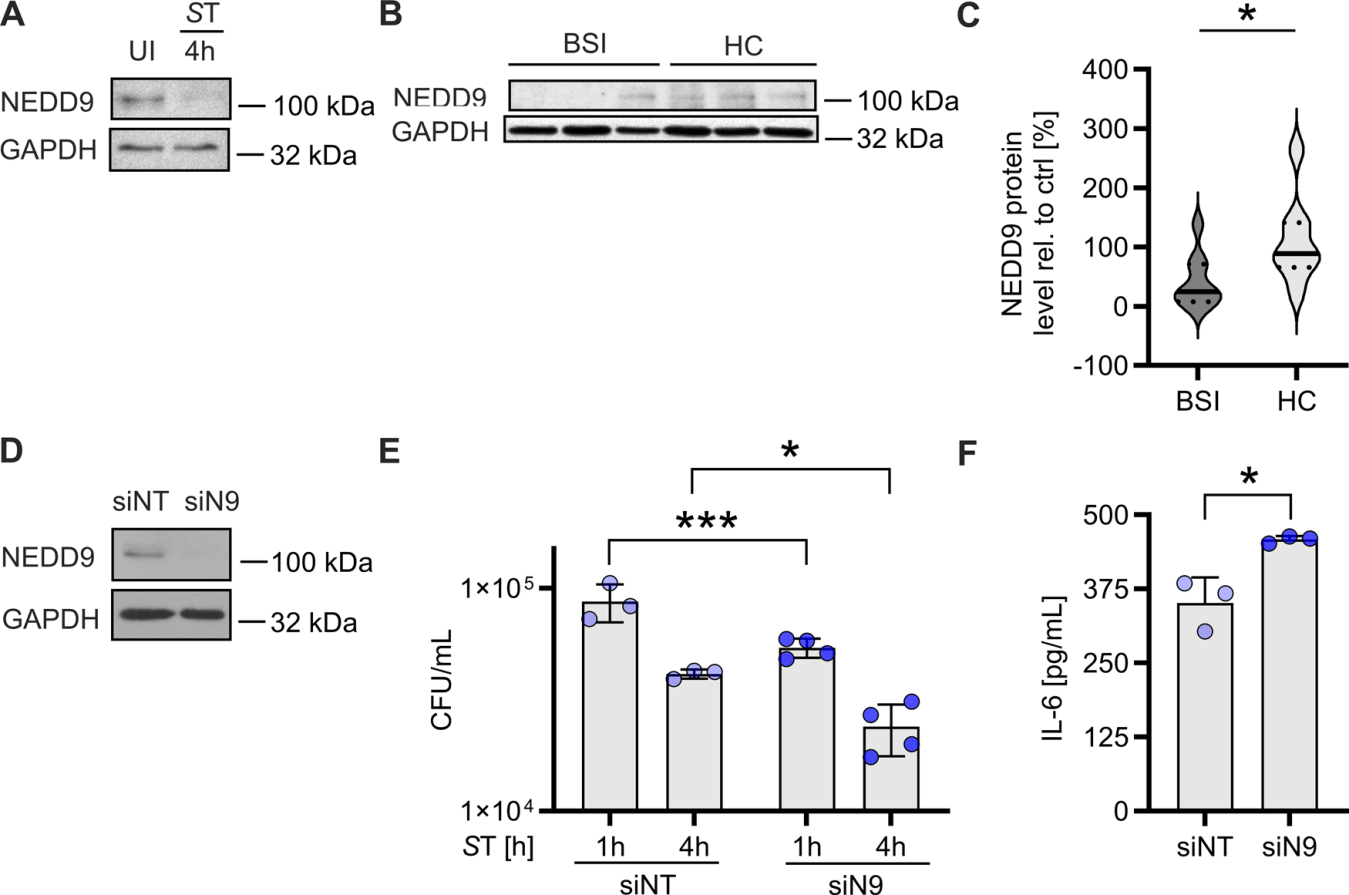
NEDD9 is downregulated in human macrophages and patients with bacterial bloodstream infections (BSIs). **A** Infection of primary human macrophages with *S*T followed by Western blot analysis showing NEDD9 protein level 4 h post infection compared to uninfected (UI) cells, n = 2. **B** Representative Western Blot analysis and (**C**) statistical analysis of NEDD9 protein expression normalized to GAPDH in PBMCs from patients suffering from bacterial BSI, *n* = 9, versus healthy control donors (HC), *n* = 8, unpaired t-test: *p* = 0.0309. **D** Primary human macrophages were transfected with non-target siRNA (siNT) or siRNA targeting NEDD9 (siN9) followed by infection with *S*T for 1 h and 4 h. Western blot analysis shows NEDD9 protein expression and (**E**) assessment of in vitro bacterial burden (CFU/mL), *n* = 4, 2-way ANOVA: *p* (siNT 1 h vs. siN9 1 h) = 0.0006, *p* (siNT 4 h vs. siN9 4 h) = 0.0272. **F** IL-6 quantification using ELISA supernatant 4 h post-infection with *S*T, unpaired t-test: *p* = 0.0130, *n* = 3. *p ≤ 0.05, ***p ≤ 0.001.

Finally, to determine whether NEDD9 downregulation and subsequent increased bacterial clearance is clinically significant, we infected human macrophages derived from healthy donors with *S*T and analyzed NEDD9 levels by Western Blot analysis. NEDD9 expression was strongly decreased in human macrophages 4 hours post *S*T infection (Fig. 6A). Next, we analyzed NEDD9 protein expression in PBMCs from patients suffering from various bacterial BSI and healthy controls (Supplemental Table S1). Consistent with our previous data, we observed that NEDD9 protein levels were significantly downregulated in patients with bacterial BSI compared to healthy controls (Fig. 6B, C). Next, we investigated the functional implications of NEDD9 loss in primary human macrophages by depleting NEDD9 using NEDD9-specific small interfering RNA followed by *S*T infection (Fig. 6D). Consistent with our previous data, NEDD9 knockdown resulted in a significantly lower bacterial burden (Fig. 6E) and increased levels of proinflammatory cytokine secretion (Fig. 6F). In conclusion, our results suggest that downregulation of NEDD9 is a host defense mechanism that could serve as a potential host-directed target to treat bacterial infection (graphical abstract).

## Discussion

Although mostly studied for its role in cancer, NEDD9 has been shown to alter lymphocyte trafficking in secondary lymphoid organs, suggesting a role for NEDD9 in regulating immune responses [20]. However, neither the relevance of NEDD9 in cells of the innate immune system nor the role of NEDD9 in the response to infection has previously been systematically investigated. In this study, we observed that NEDD9 is downregulated transcriptionally and post-translationally in macrophages upon *S*T infection. In addition, treatment of macrophages with LPS – the most abundant antigen of Gram-negative bacteria – was sufficient to reduce NEDD9 levels.

To our knowledge, regulation of NEDD9 in the context of bacterial infection has not been reported previously. Only in the context of viral infection with human T-cell leukemia/lymphoma virus and interaction with oncogenic TAX protein NEDD9 was found to be upregulated and linked to oncogenesis [36]. Indeed, in many human cancer entities NEDD9 upregulation is associated with tumor aggressiveness, metastasis and resistance to chemotherapy [17, 37, 38]. However, in a mouse model for CML, loss of NEDD9 suppressed disease progression emphasizing its cell-specific function [20]. NEDD9 levels were decreased in macrophages in a virulence-independent manner by *S*T and can even be triggered by LPS stimulation but not by TLR-2 agonist or Gram- positive bacteria such as *SA*. Therefore, we propose that Nedd9 downregulation is a macrophage response specifically to Gram-negative bacterial infections.

In most cancer cells, NEDD9 promotes biological events such as migration, invasion, epithelial mesenchymal transition (EMT) and chemotaxis. Thereby, NEDD9 is primarily located in the cytosol but translocates to different sites in the cell in a context-specific manner, including focal adhesions to increase extra-cellular matrix binding [39] or the centrosome and basal body during the cell cycle to induce mitotic spindle formation [40] or ciliary disassembly [33]. In our study, we observed that *S*T infection led to a change in the localization of NEDD9 from the cytosol to bacteria-containing phagosomes and eventually degraded in lysosomes. NEDD9 is very dynamically regulated in its function as a scaffold protein, both transcriptionally and post-translationally. In this context, NEDD9 has been shown to be degraded by various stimuli, e.g. at the end of mitosis by proteasomal degradation [29]. In contrast, in our study, inhibition of the proteasomes did not alter the degradation of NEDD9 in macrophages after infection. Instead, inhibition of the lysosomal pathway resulted in stabilization of NEDD9 and increased bacterial burden in *Nedd9*^*-/-*^ mBMDMs after *S*T infection. The fact that NEDD9 colocalized with bacteria-containing phagosomes including lysosomal marker LAMP1, as well as with pFAK upon *S*T infection and was downregulated via lysosomal degradation suggests a regulatory function in macrophage defense against bacteria. Moreover, phagolysosomal functions were significantly enhanced upon loss of NEDD9 resulting in reduced bacterial burden.

Acceleration of phagolysosomal maturation can be mediated through many different factors [41]. Secretion of cytokines is a well-established mechanism by which phagolysosomal maturation can be enhanced and ultimately support bacterial clearance [42]. NF-κb is a broad regulator not only of inflammation but also of NEDD9 transcription according to an in silico analysis of the NF-κb signaling pathway [17]. However, the interference of NEDD9 and NF-κb has not yet been further investigated. In line with the in silico analysis we found that loss of NEDD9 was associated with decreased phosphorylation of NF-κb and decreased phosphorylation of p38, whereas secretion of pro-inflammatory cytokines such as IL-6 and TNF-alpha was increased upon infection, suggesting NF-κb and p38 independent regulation of cytokine release by other pathways. This finding is consistent with previous studies showing increased secretion of pro-inflammatory cytokines and enhanced lysosomal functions despite reduced NF-κb phosphorylation, which may alternatively be mediated by mitochondrial metabolism [43]. Indeed, loss of NEDD9 not only resulted in increased release of pro-inflammatory cytokines but also in enhanced bacterial clearance, both in vitro and in vivo.

To evade host defense, we and others have shown Gram-negative bacteria such as *S*T develop an adaption mechanism by recruiting FAK and AKT at the site of *S*T-containing vacuoles to inhibit autophagy, which subsequently prevents their lysosomal degradation [7, 8]. FAK is known to be directly activated upon *S*T infection and thereby promotes bacterial survival via the AKT-mTOR signaling pathway [10]. This process is dependent on FAK kinase activity and negatively regulates autophagy via interaction with scaffolding protein p130CAS, a paralog of NEDD9 [10, 44]. We show that NEDD9 translocates to phagolysosomes upon infection where it colocalizes with FAK (Fig. 5D, E). NEDD9 is known to form a complex with kinases, in particular with FAK, to mediate their activity and regulate cellular processes such as migration, proliferation and adhesion [16, 23, 24]. As NEDD9 is recruited to *S*T-containing vacuoles we analyzed if NEDD9 is involved in the *S*T induced FAK-AKT pathway to inhibit lysosomal degradation. Indeed, our results show, that loss of NEDD9 leads to decreased FAK expression and phosphorylation in *S*T-infected macrophages. NEDD9-dependent FAK signaling has been previously reported in the context of cancer [16]. This data indicates that the presence of NEDD9 at *S*T-containing vacuoles is essential for FAK upregulation and activation upon infection, an *S*T survival mechanism to hinder their lysosomal degradation in macrophages.

Downstream of FAK, *S*T infection is known to induce phosphorylation of AKT in macrophages thereby bypassing immune defense mechanisms such as autophagy [7]. AKT is recognized as a general regulator of lysosomal function associated with bacterial infections and beyond, and is also a known downstream target of NEDD9 [8, 10]. In line with this, we observed phosphorylation of AKT and AKT substrates such as mTOR and NDRG-1 to be increased in *Nedd9*^*wt/wt*^ mBMDMs but decreased upon *Nedd9* loss, which suggests that AKT activation is dependent on NEDD9. Accordingly, bacterial load was reduced in *Nedd9*^*-/-*^ compared to *Nedd9*^*wt/wt*^ macrophages confirming previous findings that inhibition of AKT enhances bacterial clearance in macrophages. This data also corroborates with previous findings that flavonoids suppressing NEDD9 lead to lysosome-dependent downregulation of AKT [45, 46].

In addition, loss of *Nedd9* resulted in significantly increased LysoTracker staining and increased cathepsin D expression in phagosomes isolated from *Nedd9*^*-/-*^ cells as well as decreased p62 protein level, indicative for increased autophagic flux. In line with this, measuring lysosomal ß-galactosidase activity on C_12_FDG confirmed lysosomal function is enhanced in *Nedd9*^*-/-*^ BMDMs upon infection. Thus, *Nedd9* loss enhances phagolysosomal activity in macrophages and thereby counteracts the *S*T evasion mechanism of FAK-AKT activation to inhibit lysosomal function. In accordance with this, previous studies have shown that NEDD9 actively suppresses autophagy in lung cancer cells, and that autophagy is enhanced in NEDD9-deficient cells [47].

In summary, our study suggests that the downregulation of NEDD9 in macrophages serves as a host defense mechanism against Gram-negative bacterial infections. Conversely, it also indicates that *S*T and other Gram-negative pathogens exploit NEDD9 as a scaffold to recruit FAK to *S*T-containing vacuoles and to activate FAK and AKT, which in turn inhibits phagolysosomal degradation of the pathogens. However, as a host defense mechanism, macrophages degrade NEDD9 to prevent its hijacking by *S*T. This degradation restores proper phagolysosomal function, thereby enhancing bacterial clearance. Supporting this mechanism, we observed consistent downregulation of NEDD9 in PBMCs from patients with BSI, in line with our in vitro and in vivo data. Nonetheless, further studies are needed to elucidate the kinetics of NEDD9 downregulation and to explore its potential as a diagnostic marker or therapeutic target. In conclusion, our study reveals for the first time a functional link between NEDD9 and macrophage-mediated antibacterial defense (see graphical abstract), thereby expanding the current understanding of NEDD9 in immune responses. This findings suggest that targeting the NEDD9 pathway may represent a promosing host-directed strategy for the treatment of bacterial infections.

## Material and Methods

### Study approval

All animal procedures were conducted in accordance with the institutional guidelines on animal welfare and were approved by the North Rhine-Westphalian State Agency for Nature, Environment, and Consumer Protection (Landesamt für Natur, Umwelt and Verbraucherschutz [LANUV] Nordrhein-Westfalen; File no: 84-02.05.40.14.082 and 84-02.04.2015.A443) and the University of Cologne, Germany.

### Human subjects

The study involving human blood samples from healthy donors was approved by the Local Ethics Commission of the University Hospital of Cologne, Germany (File no: 08-160). Written consent was received from participants prior to inclusion in the study. All clinical investigations were conducted according to the Declaration of Helsinki principles.

### Animals

#### Mice were bred under standard conditions with ventilation system

*Nedd9* knockout mice (*Nedd9*^*-/-*^) (Seo et al., 2006) and wild type mice of the same background C57Bl/6/J (*Nedd9*^*wt/wt*^) were held under specific pathogen free conditions in a conventional animal facility. For infection animals at the age of 8–12 weeks with a balanced mix of male and female animals were used. Animal randomization or blinding was not needed as WT and knockout mice were tested.

### Pathogens

The *Salmonella enterica* Serovar Typhimurium strain (SL1344) was commercially purchased. The *S*T SPI1 mutant Δ*ssaV* and SPI2 mutant Δ*invA* were kindly provided by Ivan Dikic. Other pathogens used as controls in this study *Shigella flexneri* (ATCC 12022), *Pseudomonas aeruginosa* (ATCC 27853), *Escherichia coli* (ATCC 25922), *Staphylococcus aureus* (ATCC29213) or *Listeria monocytogenes* (EGD-e) were kindly provided by Georg Plum and are used as control strains in the routine patient diagnostics at the Institute of medical Microbiology, Immunology and Hygiene of the University Hospital Cologne. These strains are registered in the American Type Culture Collection (ATCC).

### Bacteria preparation

Bacteria from a single colony was inoculated into 5 mL BHI medium and incubated overnight at 37 °C with constant agitation. Next, 1 mL bacterial suspension was transferred into 50 mL BHI and grown until the OD600 reached 1. The bacteria concentration was estimated by plating serial dilutions on BHI agar plates.

### Cell culture and bacterial infection

Bone marrow cells were differentiated into macrophages for 7–10 days in RPMI medium supplemented with 20% L929 supernatant and 10% FBS. Mycoplasma test were regularly performed. mBMDMs were infected with specified bacteria at a MOI of 10. After infection, mBMDMs were incubated with the bacteria for 10 min at room temperature (RT) and then 30 min at 37 °C. This incubation time allowed bacteria to be internalized by macrophages. Cells were then washed with RPMI medium and incubated in medium supplemented with 50 µg/ml gentamicin. After 2 hours, the gentamicin concentration was reduced to 10 µg/ml. mBMDMs were treated with 10 µM AKT-Inhibitor VIII (Enzo lifesciences), 100 ng/ml *S*T LPS (Sigma), 100 nM Concanamycin A (Sigma), 50 µM MG132 (Selleckchem), 1 µM AKT-Activator SC79 (Selleckchem) dissolved in medium for 1 hour prior to infection. Human PBMCs were isolated from 30 mL EDTA blood using Lymphoprep (Stem Cells) and the monocytes were differentiated in RPMI containing 10% FCS and 50ng/mL M-CSF.

### *S*T phagosome isolation

*S*T was grown in BHI broth until the OD600 reached 1, and then biotinylated with EZ-link NHS-Biotin reagent (Thermo Fisher Scientific). After washing, biotinylated bacteria were incubated with siMAG Streptavidin ferrofluid (Chemicell) at 37 °C. Biotinylated *S*T bound to Streptavidin ferrofluid was then separated using a magnet and the bacteria were quantified using BHI agar plates. Subsequently, mBMDMs were infected with the biotinylated *S*T bound to the Streptavidin ferrofluid at a MOI of 10. At each time point, *S*T-containing phagosomes were isolated using equilibration and lysis buffer, as previously described for bead phagosome isolation.

### In vitro bacterial burden and ELISA

After 24 hours post-infection, mBMDMs were lysed with 1% Triton X-100, 0.01% SDS in PBS. Several dilutions of the lysate were plated on BHI plates and incubated overnight at 37 °C. The next day, *S*T CFUs were enumerated. Supernatants were collected and analyzed for IL-6 and TNF-α secretion using ELISA kits (R&D, DY406-05, DY410-05), according to the manufacturer’s instructions.

### Estimation of bacterial burden in vivo

Mice were infected (i.p. injection) with 100 CFU *S*T. After 4 days of infection, mice were euthanized. The spleens were isolated, weighted and homogenized using a gentleMACS Dissociator (Miltenyi Biotec, Bergisch Gladbach, Germany) in sterile PBS. Extracts of the homogenized spleens were plated on BHI Agar plates. After 24 h incubation at 37 °C, the bacterial colonies were enumerated. The number of colonies was normalized to per gram of tissue. Spleen extracts were also used for ELISA (described above).

### Tissue histology

Spleen and liver samples were obtained from in vivo experiments and cross-sectioned in equal-sized slices 2 mm in width. The respective slices were snap-frozen in liquid nitrogen covered with Tissue-Tek® and stored at −80 °C until further use. Cryostat sections were cut to 3 µm and attached to adhesive glass slides (Dako, Hamburg, Germany), fixed with 4% phosphate-buffered formalin for 30 sec, and stained with H&E. After rinsing in water, the slides were dehydrated via an ascending ethanol gradient, immersed in xylene and mounted on glass coverslips. The tissue sections were imaged using a Hitachi HV-F202 UXGA 3CCD camera attached to Leica DM5500B microscope. Pictures were generated using the DISKUS program (Hilgers Technisches Büro, Königswinter, Germany).

### Western blotting

mBMDMs were lysed in RIPA buffer supplemented with a 1X protease/phosphatase inhibitor cocktail (Thermo Fisher Scientific). Protein concentrations were estimated using Pierce® BCA Protein Assay Kit (Thermo Fisher Scientific), according to the manufacturer’s instructions. Equal amounts of protein were separated on 10% or 12% SDS-PAGE gels or on 4–20% Mini-PROTEAN® TGX™ Precast Protein Gels (Biorad #4561094) using a broad range, color-coded prestained protein marker (#14208 Cell Signaling). The proteins were then transferred onto a PVDF membrane and probed with the following antibodies: HEF1/Nedd9 (#4044 Cell Signaling), phospho-NF-κB p65 Ser536 (#3033 Cell Signaling), NF-κb p65 (#4764 Cell Signaling), Lamp-1 (sc-17768 Santa Cruz Biotechnology), phospho-Akt Ser473 (#4060 Cell Signaling), phospho-Akt Thr 308 (#2965 Cell Signaling), Akt (#4691 Cell Signaling), phospho-FAK Tyr397 (#3283 Cell Signaling), FAK (#3285S Cell signaling), phospho-mTOR Ser2448 (#4561094 Cell Signaling), phospho-p70 S6 Kinase Thr389 (#9234#9205 Cell Signaling), phospho-NDRG-1 (#3217 Cell Signaling), p62 (#5114 Cell Signaling), LC-3 (L7543 Sigma), phospho-P38 (Cell Signaling), Cathepsin D (#2284 Cell Signaling), β-actin (A2228 Sigma-Aldrich) and GAPDH (#2118S Cell Signaling). GAPDH and ß-Actin was used as a loading control. After incubation with secondary HRP-conjugated antibodies (R&D), the blots were developed using ECL reagent (GE Healthcare). Densitometric analysis of Western Blots was performed using lab image 1D software (Kapelan Bio-Imaging GmbH, Leipzig, Germany).

### Immunofluorescence staining and confocal microscopy

mBMDMs and human macrophages grown on glass coverslips were infected with *S*T and fixed with 4% formaldehyde or methanol in PBS for 15 min at RT. When indicated, the cells were treated with LysoTracker before fixation at the indicated time points. The samples were then permeabilized with 0.3% Triton X-100 in PBS for 5 min and then incubated with Image-iT® R FX (#I36933, Invitrogen) and blocked with 5% (w/v) normal goat serum (Life Technologies). The cells were then incubated overnight with specific primary antibodies NEDD99 (#4044S, cell signaling), phospho-FAK Tyr397 (#3283 Cell Signaling), Lamp-1 (sc-17768 Santa Cruz Biotechnology) and *S*T LPS (MA1-83451, Thermo Fisher Scientific) at 4 °C. Alternatively, PE-labeled NEDD9 antibody was used (Novus NB100-1699PE). After washing with 0.03% Triton in PBS, the cells were incubated with either Alexa Fluor 594-conjugated goat anti-rabbit or anti-mouse secondary antibody or Alexa Fluor 488-conjugated goat anti-rabbit or anti-mouse (Life Technologies) for 1 hour at RT protected from light. The cover-slips were then mounted using ProLong® Gold antifade DAPI (#P36953, Life Technologies) to stain the nuclei. The slides were imaged using a 63X objective under a confocal microscope (Leica SP8, Leica Microsystems).

Colocalization was analyzed using Colocalization Analysis plugins (ImageJ software). First, the Colocalization Test plugin with Fay randomization method was performed to calculate Pearson’s correlation coefficient for the two channels in each selected ROI (25 × 25 pixels). This value was compared with what would be expected for random overlap. The observed correlation was considered significant if it was greater than 95% of the correlations between channel 1 and a number of randomized channel 2 images. All ROIs with Pearson’s coefficient *p* value of ≥ 0.95 were further analyzed by the Colocalization Threshold plugin to calculate thresholded Mander’s coefficients [tM1 colocalization value for channel 1 (red); tM2 colocalization value for channel 2 (green or blue)] and to generate scatterplots with linear regression line and thresholds.

### NEDD9 siRNA knockdown in human macrophages

Human macrophages were transfected with either 50 nM non-targeting siRNA (#SR-CL000-005, Eurogentec) or siRNA for NEDD9 (Dharmacon) using the transfection reagent Lipofectamine 3000 (Life Technologies), according to the manufacturer’s instructions.

### RNA isolation, reverse transcription and qPCR

RNA was isolated using RNeasy® Mini Kit followed by reverse transcription using GoScript™ Reverse Transcription System according to the manufacturer’s protocol. Quantitative real-time PCR for *Nedd9* was run on a 7500 Fast cycler system (Applied Biosystems, Foster City, California, United States) *Nedd9* mRNA expression was determined using TaqMan™ Fast Advanced Master Mix. GAPDH was used as a reference gene to normalize NEDD9 expression.

### Gene expression array

Total RNA was extracted from uninfected or *S*T-infected (2 hours) *Nedd9*^*-/-*^ mBMDMs using the RNeasy Mini Kit (Qiagen), and subsequently treated with RNase-free DNase I for 30 min at 37 °C to remove residual DNA. Library preparation was carried out according to Illumina’s protocol for ‘Preparing Samples for Sequencing of mRNA’ (Part #1004898, Rev. A). Briefly, mRNA was isolated from total RNA with oligo(dT) magnetic beads. Upon purification and chemical fragmentation, cleaved RNA was copied into first strand cDNA using reverse transcriptase and random primers. Second strand cDNA was synthesized using DNA Polymerase I and RNase H. Following end repair, addition of a single A base, and adaptor ligation, cDNA was size-selected on agarose gels and products were further purified and enriched by PCR amplification to create the final cDNA library. Library integrity was analyzed using an Agilent Technologies 2100 Bioanalyzer and libraries were subsequently sequenced on an Illumina HiSeq 2000 platform (BGI Genomics, China) (GSE84375) [25].

### Quantification of hydrolase activity

1 × 10^8^ ST in 100 µl PBS were labeled with 350 µg/ml 5-dodecanoylaminofluorescein-di-β-D-galactopyranoside (C_12_FDG, D2893, Thermo Fisher Scientific) in 0.1 M NaHCO_3_ buffer, pH 9.6 for 1 hour at 37 °C on a tube rotator. mBMDMs (50,000 cells/well) were infected at a MOI of 50 in a 96-well black flat bottom plate and then incubated at 37 °C in HBSS with Ca2^+^ and Mg2^+^ in a Tristar2 multimode plate reader LB 942 (Berthold Technologies). Unquenching of C_12_FDG fluorescence was measured at 120 s intervals using standard GFP filters. Phagosomal acidification, and thereby acid hydrolase activity, was inhibited by 5 µM chloroquine.

### Bioinformatic analyses

Raw sequencing reads were filtered into clean reads and were aligned to murine reference sequences (ftp://ftp.ncbi.nih.gov/refseq/M_musculus/mRNA_Prot/) using SOAPaligner/SOAP2 [48]. Gene expression values were then calculated by the RPKM method [49] and differentially expressed genes (DEGs) among uninfected and *S*.Typhimurium-infected (2 hours) *Nedd9*^*-/-*^ mBMDMs were identified. Up and downregulation of single genes was assessed by calculating the log2 of fold changes (log2[RPKM WT ST/RPKM WT]). Correction for false positive (type I errors) and false negative (type II) errors was performed based on the false discovery rate (FDR) method [50]. ‘FDR ≤ 0.001 and log2 ratio ≥ 1’ were set as the cutoff to determine the significance of DEGs.

### Statistical Analysis

Statistical analysis and data evaluation were carried with GraphPad Prism version 10.2.3 for Windows, GraphPad Software, La Jolla, California, United States. Data are presented as mean ± SD if not otherwise indicated. Significance is indicated directly in the figures as *p ≤ 0.05, **p ≤ 0.01, ***p ≤ 0.001, ****p ≤ 0.0001. Unpaired t-test was performed to compare two independent groups with normally distributed data. Data of two independent groups that are not normally distributed were compared by using Mann-Whitney test. One-way ANOVA was used to compare three or more independent groups for one single factor and Kruskal-Wallis test was used when assumptions of ANOVA were not met. Two-way ANOVA was used to determine the effect of two different independent factors.

## Supplementary information

Supplementary Figures

Supplementary Table 1

uncropped westernblots qRT PCR

## Data Availability

All data generated or analysed during this study are included in this published article.
