# Correction: Macrophages downregulate NEDD9 to counteract *S*. Typhimurium- mediated FAK-AKT activation and lysosome inhibition

**DOI:** 10.1038/s41419-025-07919-z

**Published:** 2025-08-29

**Authors:** Julia Fischer, Lisa Rusyn, Frederike Krus, Liudmila Lobastova, Marc Herb, Alexander Gluschko, Zahra Hejazi, Nina J. Hos, Chiara Calabrese, Jannik Stemler, Petra Mayer, Ruth Hanssen, Sebastian J. Theobald, Jörg Janne Vehreschild, Jonel Trebicka, Martin Krönke, Jochen W. U. Fries, Clara Lehmann, Phuong-Hien Nguyen, Jan Rybniker, Nirmal Robinson, Tamina Seeger-Nukpezah

**Affiliations:** 1 University of Cologne, Faculty of Medicine and University Hospital of Cologne, Department I of Internal Medicine, Center for Integrated Oncology, Cologne, Germany; 2 University of Cologne, Faculty of Medicine and University Hospital of Cologne, Center for Molecular Medicine Cologne (CMMC), Cologne, Germany; 3 German Center for Infection Research (DZIF), Partner Site Bonn-Cologne, Cologne, Germany; 4 University of Münster, Faculty of Medicine and University Hospital of Münster, Department of Internal Medicine B, Albert-Schweitzer-Campus, Münster, Germany; 5 Cologne Excellence Cluster on Cellular Stress Responses in Aging-Associated Diseases (CECAD), Chair Translational Research, Faculty of Medicine and University Hospital Cologne, University of Cologne, Cologne, Germany; 6 University of Cologne, Faculty of Medicine and University Hospital of Cologne, Institute of Medical Microbiology, Immunology and Hygiene, Cologne, Germany; 7 Department of Clinical Research, Clinic of Infectious Diseases, London School of Hygiene and Tropical Medicine, WC1E 7HT, London, United Kingdom; 8 Max Planck Institute for Biology of Ageing, Cologne, Germany; 9 Department II of Internal Medicine, Hematology/Oncology and Infectious Diseases, University Hospital of Frankfurt, Frankfurt, Germany; 10 University of Cologne, Faculty of Medicine and University Hospital of Cologne, Policlinic for Endocrinology, Diabetes and Preventive Medicine, Cologne, Germany; 11 Max Planck Institute for Metabolism Research, Cologne, Germany; 12 European Foundation for the Study of Chronic Liver Failure - EF Clif, Barcelona, Spain; 13 Institute for Bioengineering of Catalonia, Barcelona, Spain; 14 Department of Medical Gastroenterology and Hepatology, University of Southern Denmark, Odense, Denmark; 15 University of Cologne, Faculty of Medicine and University Hospital of Cologne, Institute of Pathology, University of Cologne, Cologne, Germany; 16 Center for Cancer Biology, University of South Australia and SA Pathology, Adelaide, SA Australia; 17 Adelaide Medical School, Faculty of Health and Medical Sciences, The University of Adelaide, Adelaide, SA Australia

**Keywords:** Infection, Infection

Correction to: *Cell Death & Disease* 10.1038/s41419-025-07634-9, published online 12 June 2025

In this article the author name Sebastian J. Theobald has been initally written Sebastian Theobald.

The original article has been corrected.

