# Supplementary material for: Macrophages downregulate NEDD9 to counteract S. Typhimurium- mediated FAK-AKT activation and lysosome inhibition
Source: Cell Death Dis. 2025 Jun 12;16(1):445. doi: 10.1038/s41419-025-07634-9 (PMC12162842; doi:10.1038/s41419-025-07634-9)
Supplement: Supplementary file 1 — Supplementary Figures [file 41419_2025_7634_MOESM1_ESM.pdf]

Figure S1

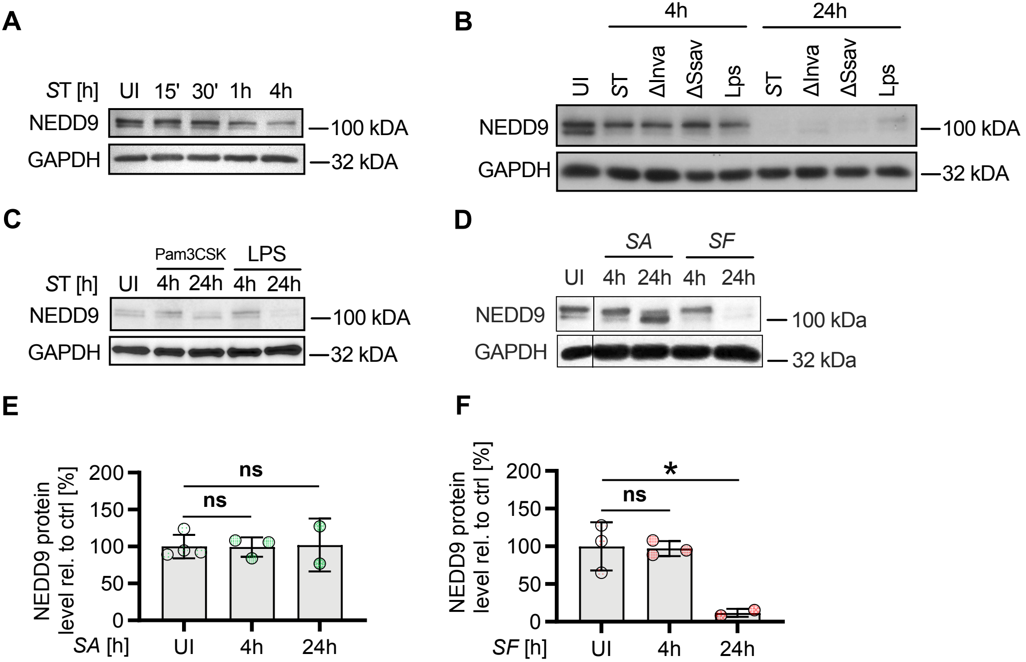

Figure S1. NEDD9 is downregulated upon infection in a virulence-independent manner.
(**A**) mBMDMs infected with *S*T for indicated time points and blotted for NEDD9 in Western blot analysis n = 1. (**B**) mBMDMs were infected with *S*T variants for 4h and 24h n = 1. (**C**) mBMDMs treated with PAM3SK or LPS 100ng/ml for 4h and 24h and NEDD9 level was analyzed by Western Blotting, n = 2. (**D**) Western blot of mBMDMs infected with SA and SF at indicated time points. (**E**) Densitometric analysis of NEDD9 protein level normalized to GAPDH upon SA infection, Kruskal-Wallis test: no significance and (**F**) SF infection, n = 3, biological replicates, one-way ANOVA: p (UI vs. 24h) = 0.0109.
A p-value > 0.05 equals not significant (ns), * = p ≤ 0.05, ** = p ≤ 0.01, *** = p ≤ 0.001, **** = p ≤ 0.0001.

Figure S2

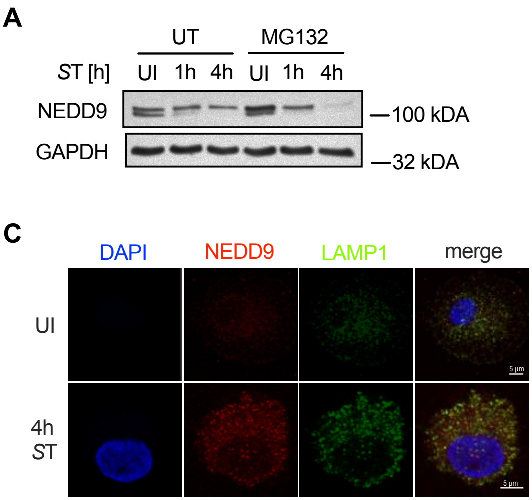

Figure S2. Increased phagolysosomal activity upon loss of NEDD9.
(**A**) Infection of *Nedd9*$^{wt/wt}$ mBMDMs with *ST* and parallel treatment with MG132 (50µM) followed by Western blotting, n = 3, biological replicates. (**B**) Infection of *Nedd9*$^{wt/wt}$ mBMDMs with *ST* followed by immunofluorescence staining of *ST* (blue), NEDD9 (red) and LAMP1 (green), n = 10, biological replicates. (**C**) Representative confocal microscopy images of human macrophages uninfected and infected for 4 hours with *ST* and stained for NEDD9 (red) and LAMP1 (green).

Figure S3

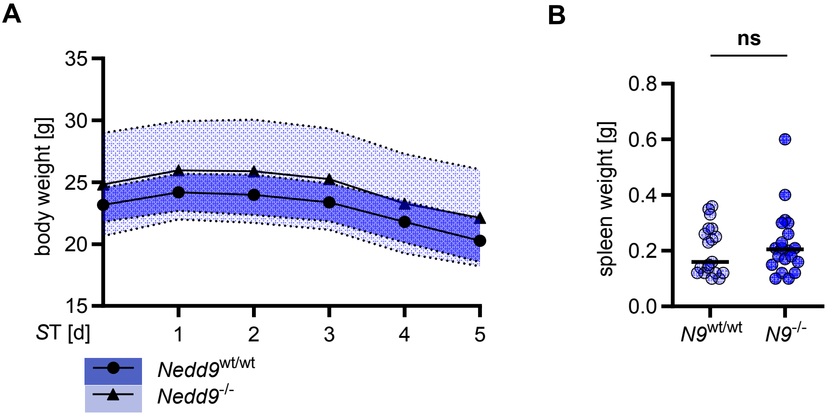

Figure S3. Bodyweight reduction and spleen weight are not affected by the loss of NEDD9. Infection of *Nedd9*$^{wt/wt}$ and *Nedd9*$^{-/-}$ mice with *S*T for 4 days. (**A**) Bodyweight of *Nedd9*$^{wt/wt}$ and *Nedd9*$^{-/-}$ mice throughout *S*T infection. *Nedd9*$^{wt/wt}$ n = 6, *Nedd9*$^{-/-}$ n = 8, biological replicates. (**B**) Spleen weight of *Nedd9*$^{wt/wt}$ and *Nedd9*$^{-/-}$ mice 4 days post *S*T infection. *Nedd9*$^{wt/wt}$ n = 19, *Nedd9*$^{-/-}$ n = 20, biological replicates, Mann-Whitney test: no significance.

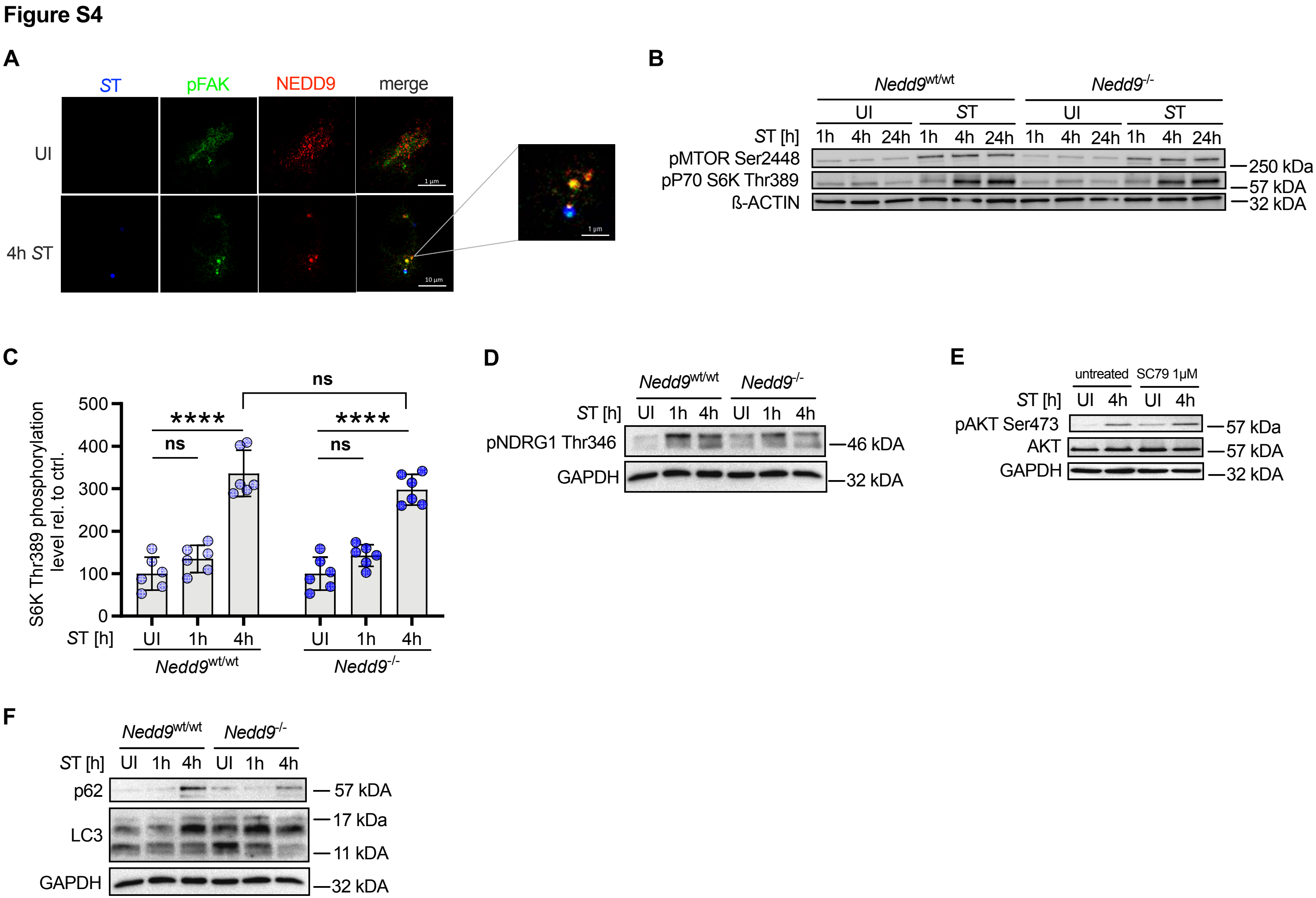

Figure S4. Increased phagolysosomal capacities by reduced mTOR/p62 signaling.
(**A**) IF Triplestaining of pFAK, NEDD9 and *ST* in *Nedd9*$^{wt/wt}$ mBMDMs uniinfected and ST4h. (**B**) Western blot analysis of phospho-mTOR and phospho-S6K, n = 3, biological replicates. (**C**) Densitometric analysis of Western blots showing phosphorylated S6 kinase (S6K), 2-way ANOVA: p (*Nedd9*$^{wt/wt}$ UI vs. *ST* 4h) < 0.0001, p (*Nedd9*$^{-/-}$ UI vs. *ST* 4h) < 0.0001. (**D**) Western blot analysis of phospho-NDRG-1, n = 3, biological replicates. (**E**) Western blot analysis of *ST* infected *Nedd9*$^{-/-}$ mBMDMs untreated and treated for 1h and 4h with 1µM AKT activator SC-79 and blotted for pAKTSer473 and total AKT, n = 3, biological replicates. (**F**) Western blot analysis of p62 and LC3, n = 3, biological replicates.
