# Supplementary material for: Macrophages downregulate NEDD9 to counteract S. Typhimurium- mediated FAK-AKT activation and lysosome inhibition
Source: Cell Death Dis. 2025 Jun 12;16(1):445. doi: 10.1038/s41419-025-07634-9 (PMC12162842; doi:10.1038/s41419-025-07634-9)
Supplement: Supplementary file 2 — Supplementary Table 1 [file 41419_2025_7634_MOESM2_ESM.pdf]

**Characteristics**<br>**n = 17**	**BSI**<br>**n = 9 (%)**	**Healthy controls**<br>**n = 8 (%)**
**Sex**		
Male	3 (33,33)	3 (37,5)
Female	6 (67,77)	5 (62,5)
**Age (years)**		
Range	25 – 81	21- 76
Mean	58	45
**Pathogen**		
Enterococcus faecium	1	
Escherichia coli	4	
Klebsiella pneumoniae	2	
Pseudomonas aeruginosa	1	
Staphylococcus aureus	2	
**Day of BSI onset**		
Range	1-5d	
Mean		
