# Supplementary material for: Macrophages downregulate NEDD9 to counteract S. Typhimurium- mediated FAK-AKT activation and lysosome inhibition
Source: Cell Death Dis. 2025 Jun 12;16(1):445. doi: 10.1038/s41419-025-07634-9 (PMC12162842; doi:10.1038/s41419-025-07634-9)
Supplement: Supplementary file 3 — uncropped westernblots qRT PCR [file 41419_2025_7634_MOESM3_ESM.pdf]

Uncropped WB for manuscript CDDIS-24-5020

Fig.1 D

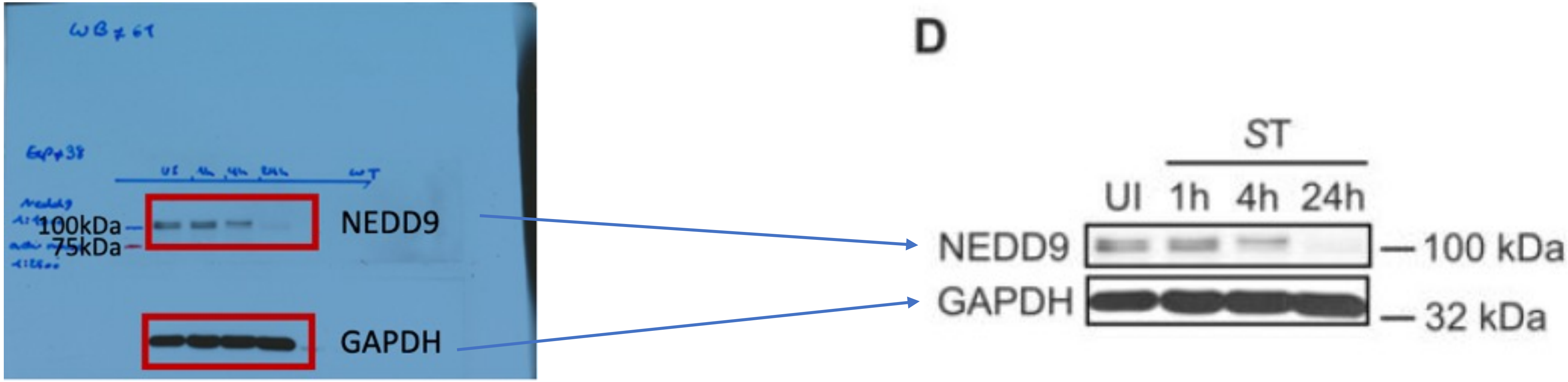

100kDa
75kDa
NEDD9
GAPDH
D
ST
UI
1h
4h
24h
NEDD9
GAPDH
100 kDa
32 kDa

Fig.2 C

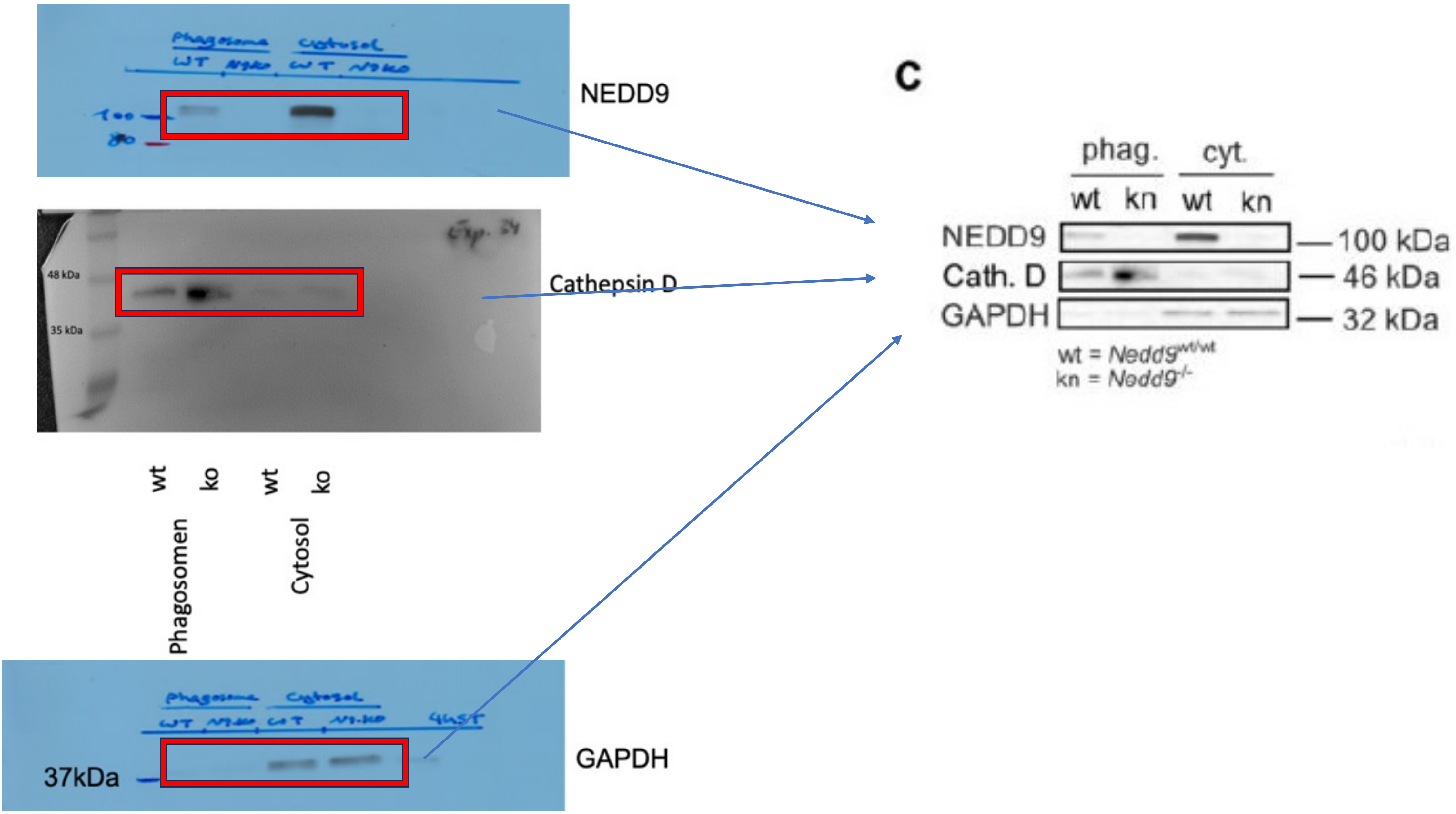

NEDD9
Cathepsin D
GAPDH
48 kDa
35 kDa
37kDa
wt
ko
wt
ko
Phagosomen
Cytosol
C
phag.
cyt.
wt
kn
wt
kn
NEDD9
Cath. D
GAPDH
100 kDa
46 kDa
32 kDa
wt = Nedd9wt/wt
kn = Nedd9-/-

Fig.2 D

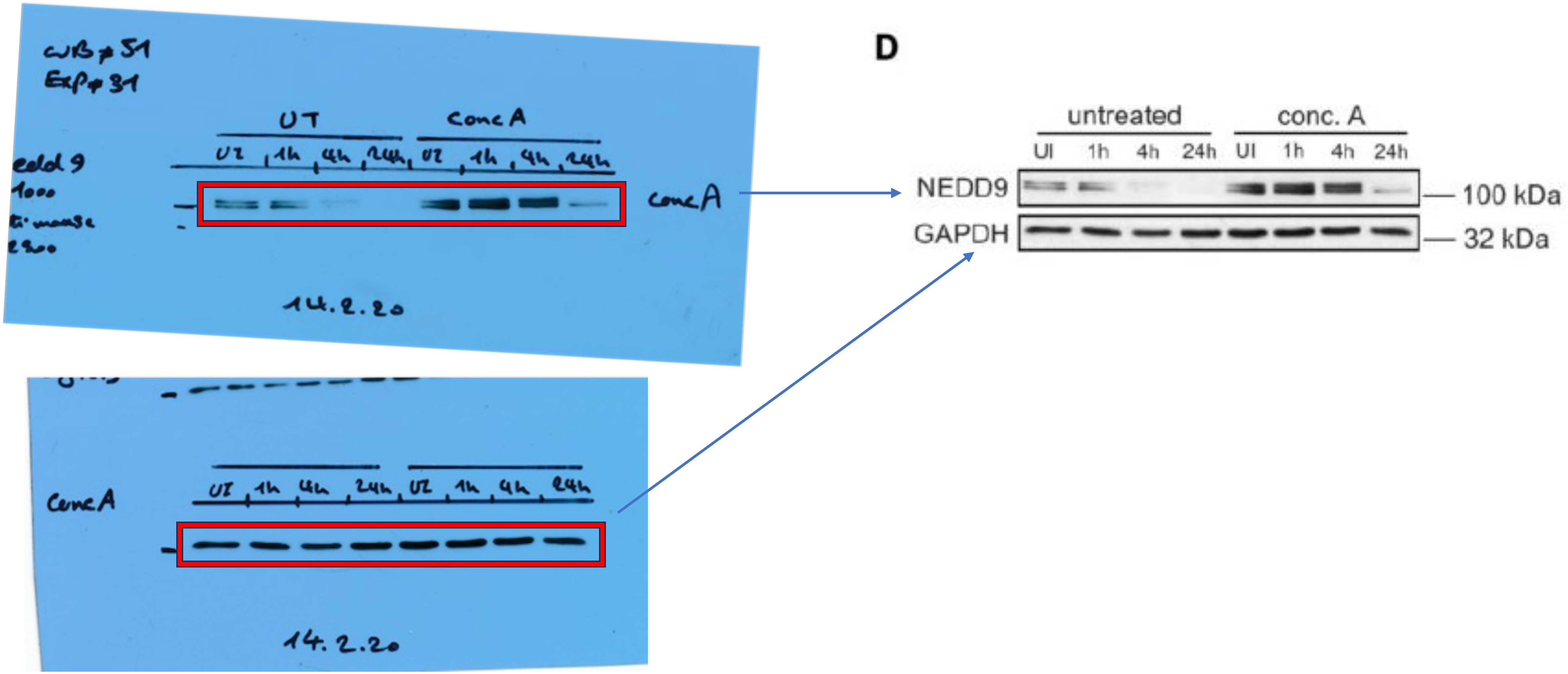
WB # 51
Exp # 31
UT
Conc A
UI 1h 4h 24h UI 1h 4h 24h
Conc A
14.2.20
Conc A
UI 1h 4h 24h UI 1h 4h 24h
14.2.20
D
untreated
conc. A
UI 1h 4h 24h UI 1h 4h 24h
NEDD9
GAPDH
100 kDa
32 kDa

Fig.3C

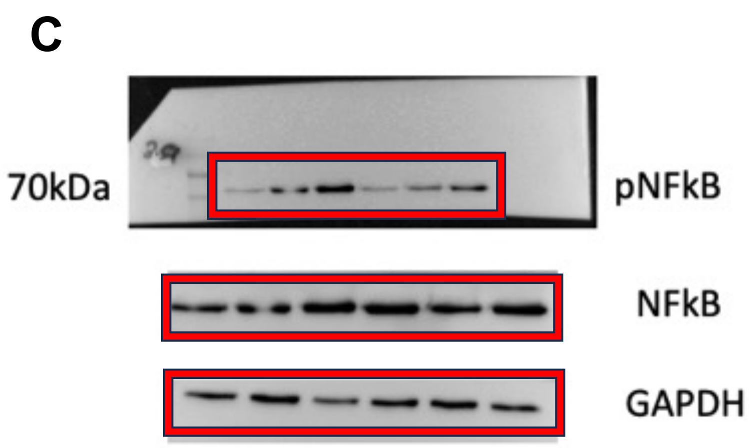
C
70kDa
pNFkB
NFkB
GAPDH

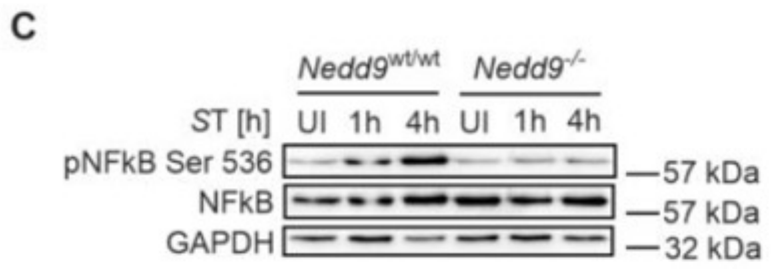
C
Nedd9wt/wt
Nedd9-/-
ST [h]
UI 1h 4h UI 1h 4h
pNFkB Ser 536
NFkB
GAPDH
57 kDa
57 kDa
32 kDa

Fig. 3 D

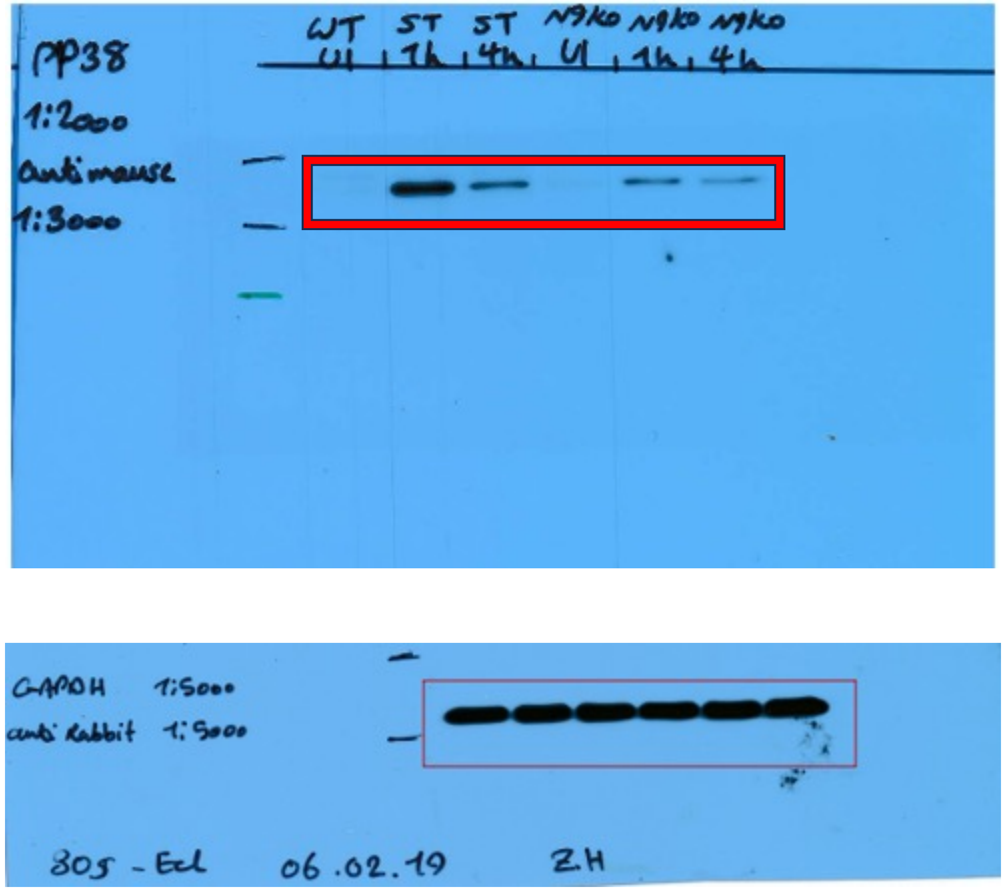

pP38
1:2000
anti mouse
1:3000
WT UI
ST 1h
ST 4h
N9KO UI
N9KO 1h
N9KO 4h
GAPDH 1:5000
anti Rabbit 1:5000
805 - Ecl
06.02.19
Z.H

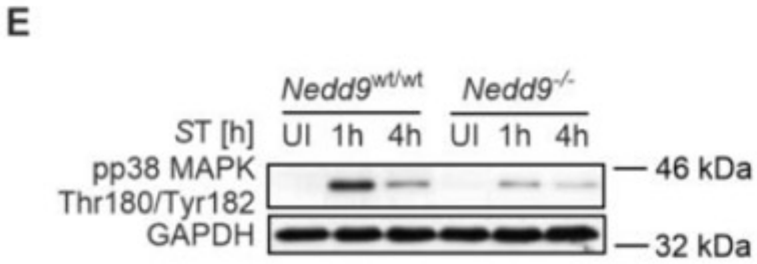

E
Nedd9wt/wt
Nedd9-/-
ST [h]
UI
1h
4h
UI
1h
4h
pp38 MAPK
Thr180/Tyr182
GAPDH
46 kDa
32 kDa

Fig.5 A

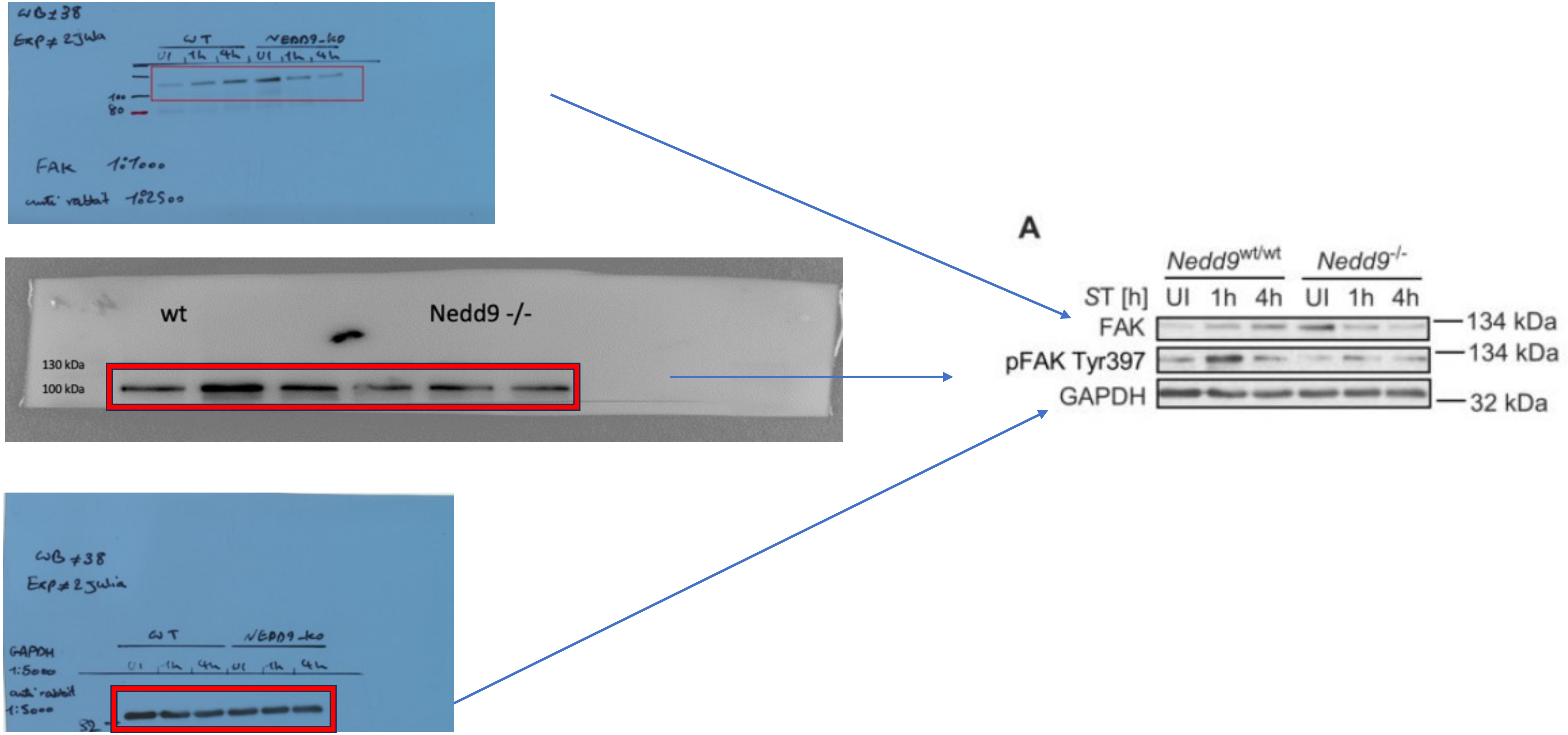

WB#38
WT
NEDD9-ko
UI 1h 4h UI 1h 4h
100
80
FAK 1:1000
anti rabbit 1:2500
wt
Nedd9 -/-
130 kDa
100 kDa
WB#38
GAPDH
1:5000
anti rabbit
1:5000
32
A
Nedd9wt/wt
Nedd9-/-
ST [h] UI 1h 4h UI 1h 4h
FAK
pFAK Tyr397
GAPDH
134 kDa
134 kDa
32 kDa

Fig.5 E

Fehlen total AKT u. GAPDH

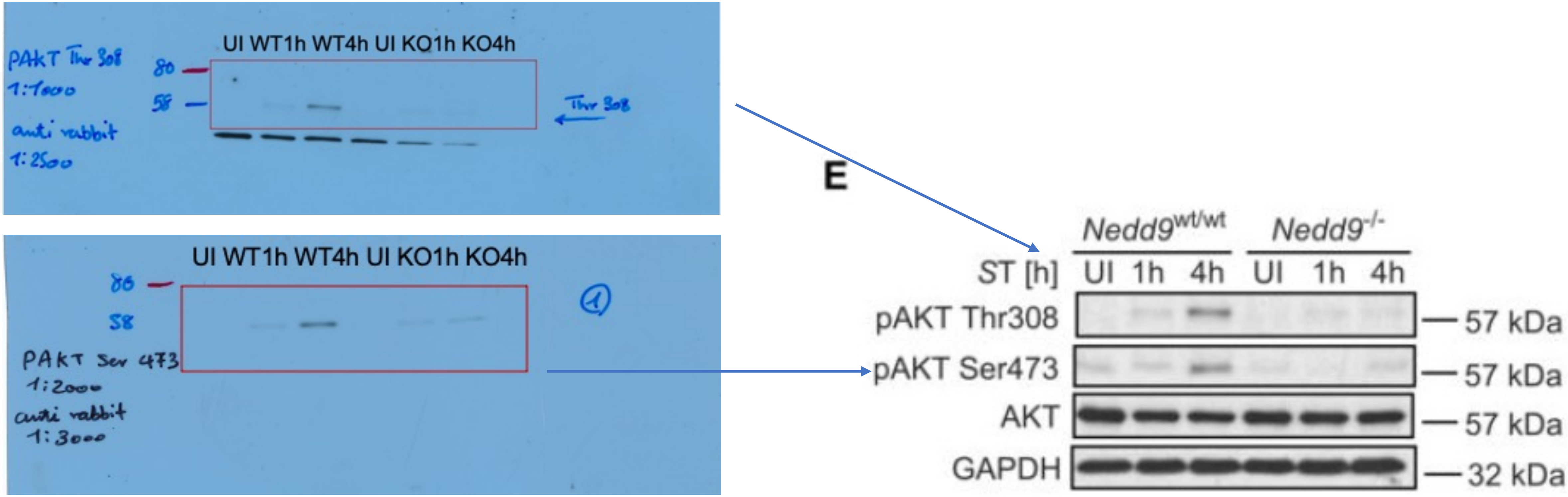

Fig.6 A

NEDD9

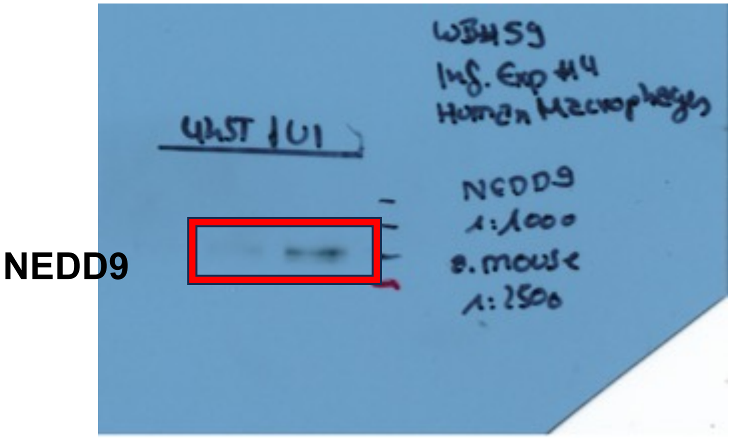

Flipped horizontally

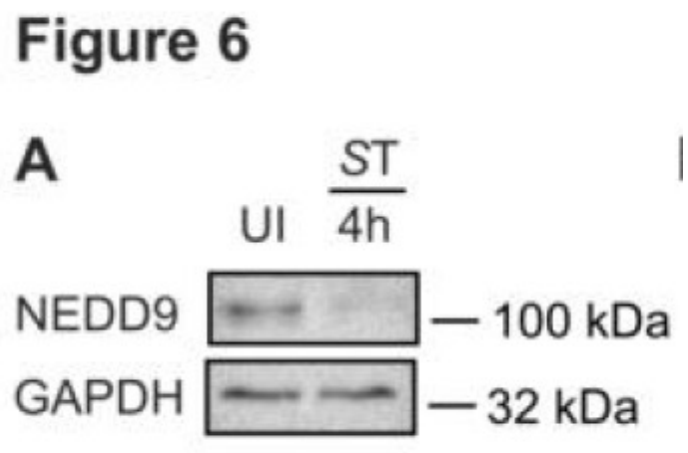

GAPDH

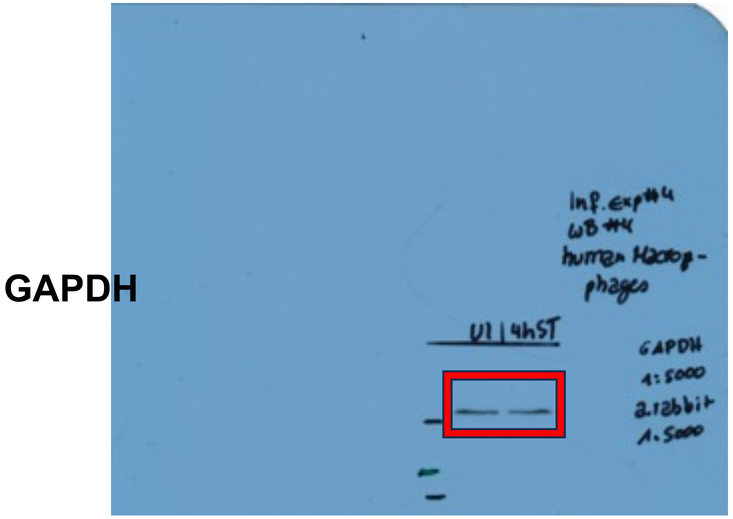

Fig.6 B

Fehlen GAPDH

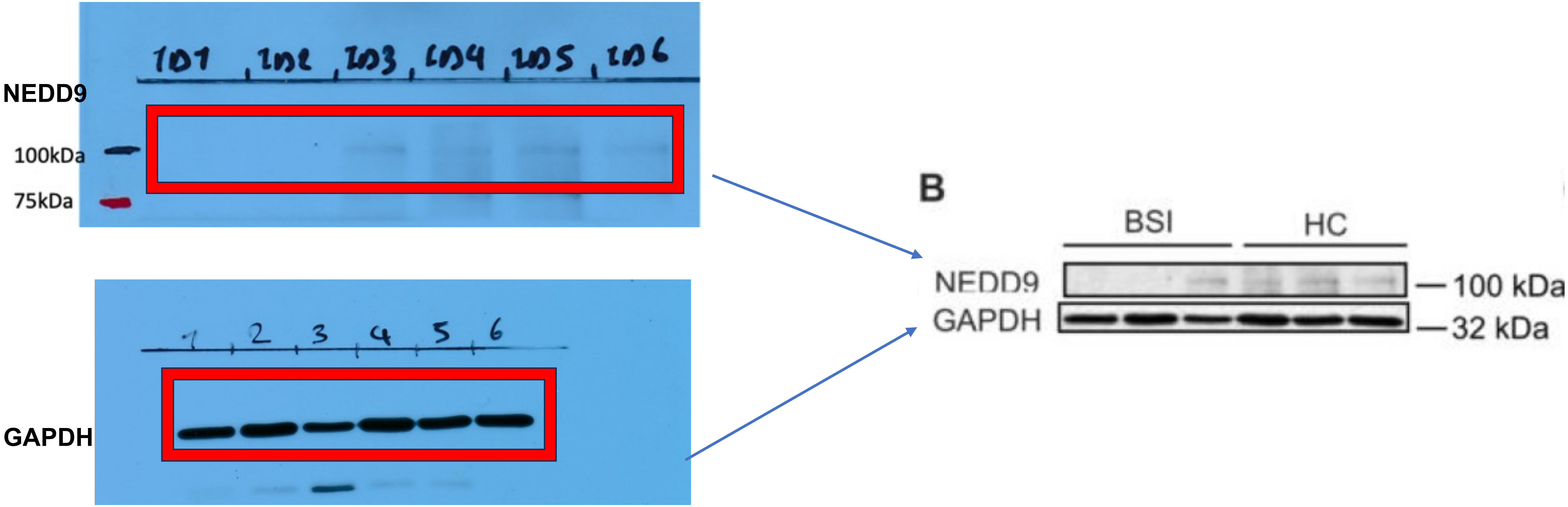

Fig.6 D

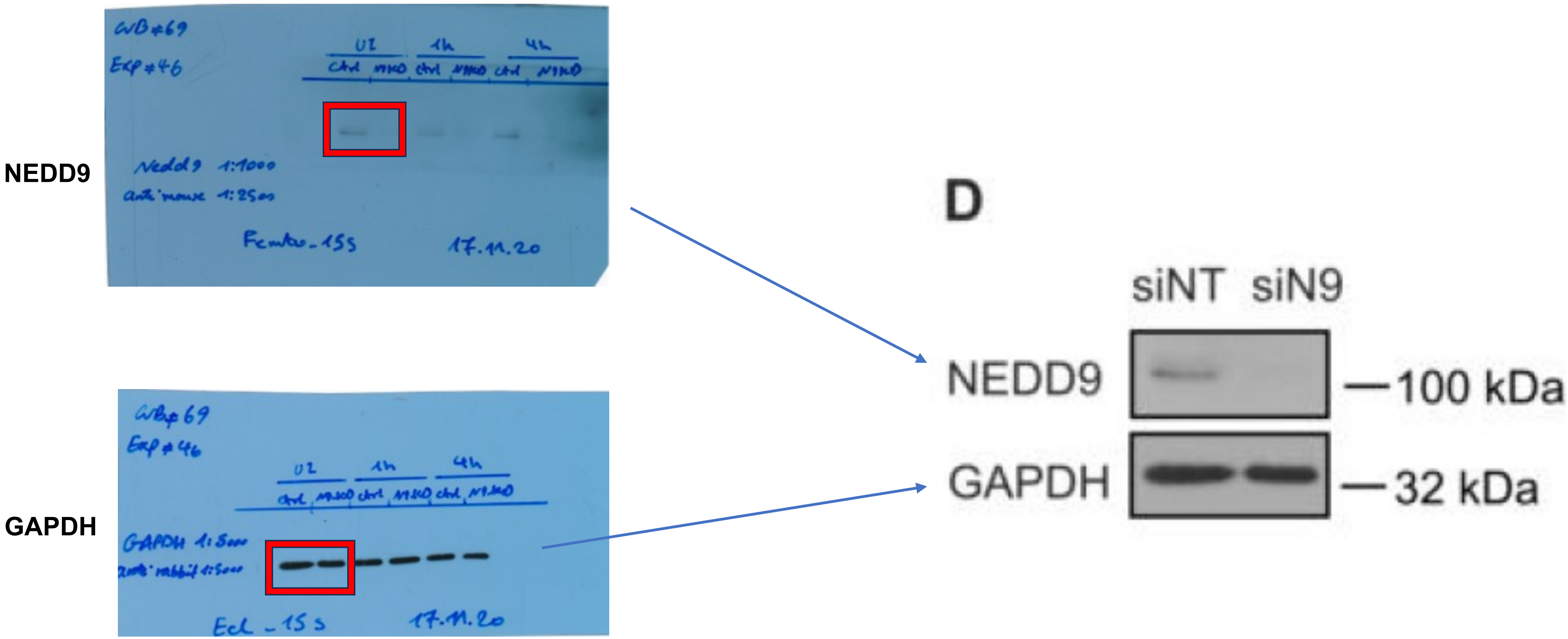

Fig.S1 A

NEDD9

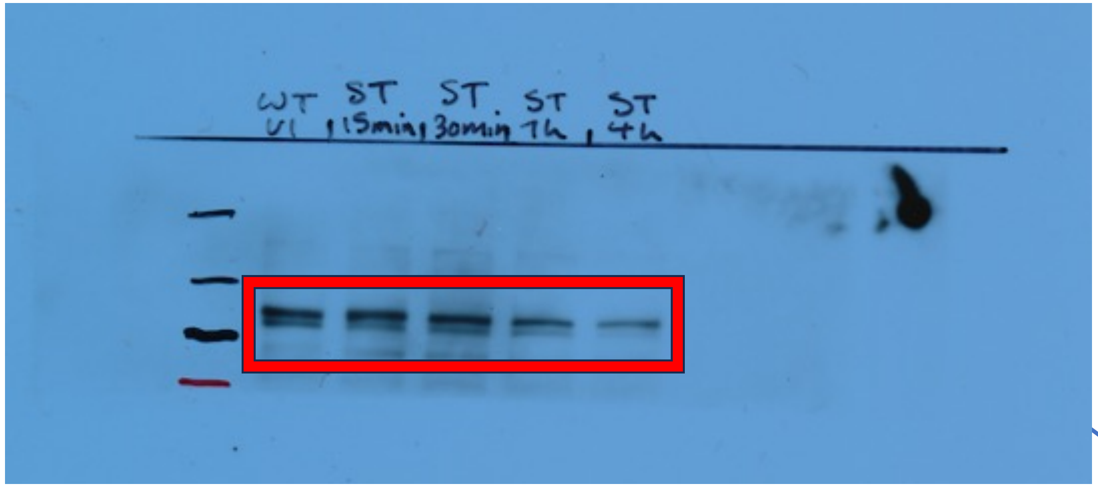

GAPDH

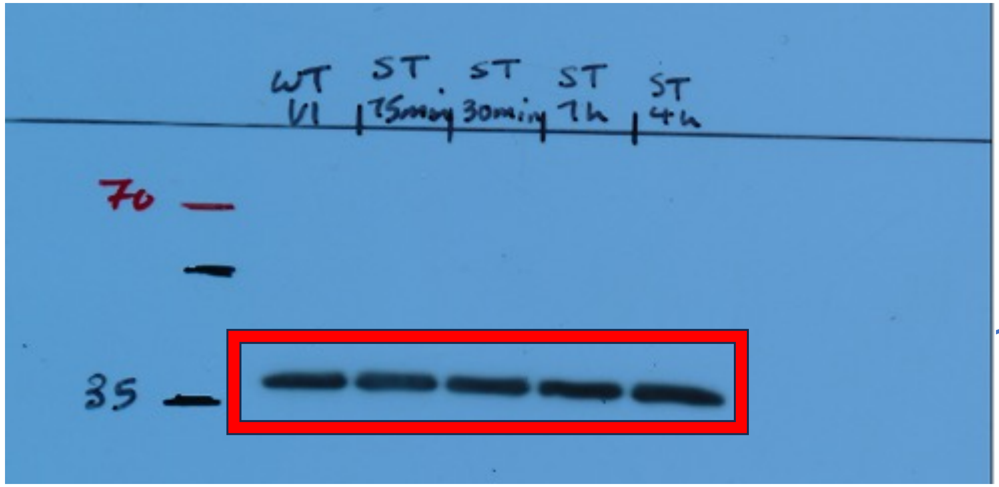

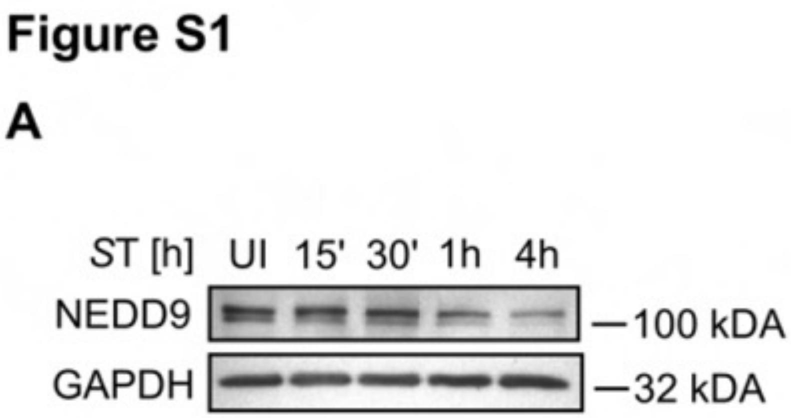

Fig.S1 B

NEDD9

GAPDH

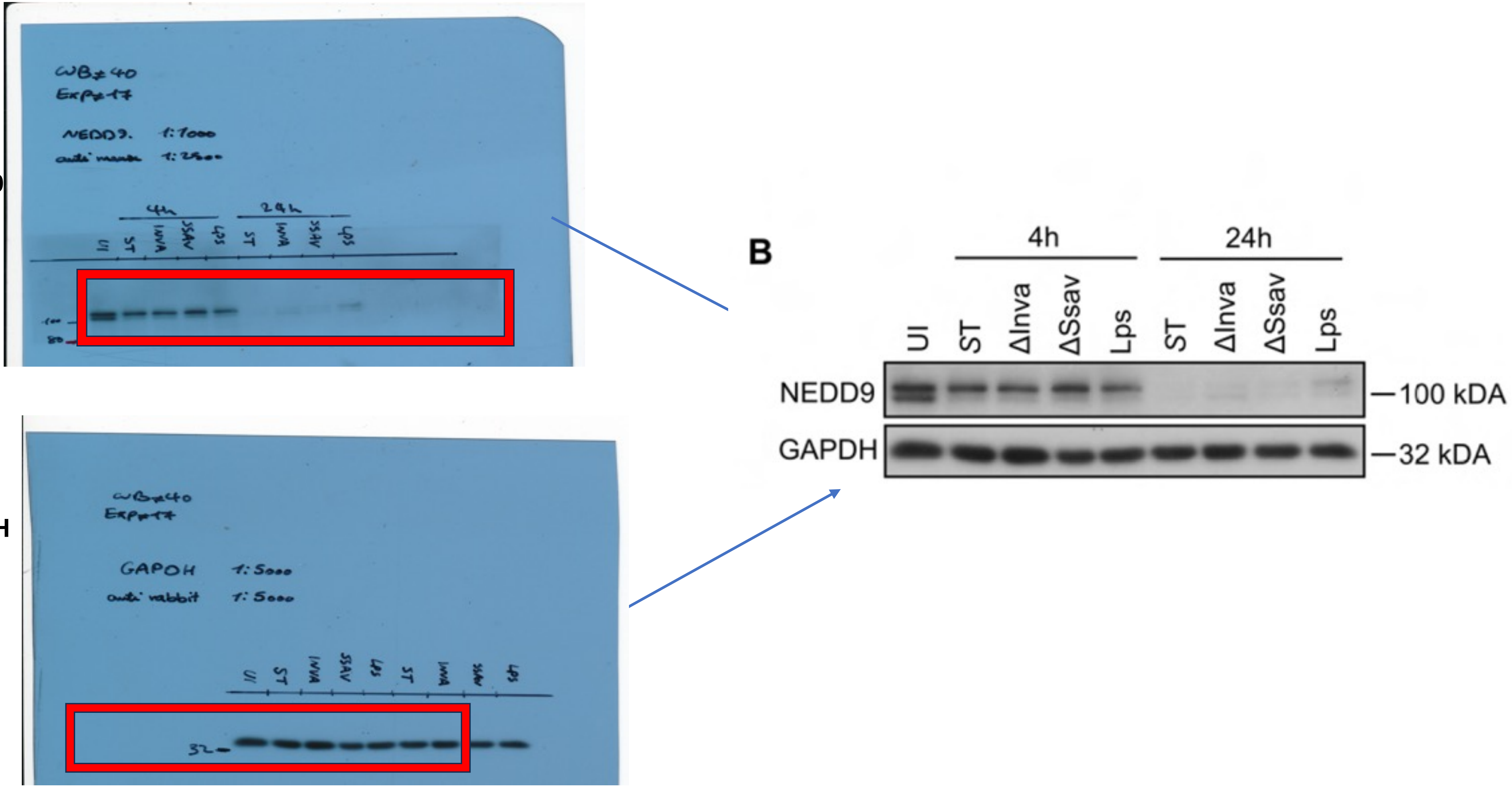

Fig.S1 C

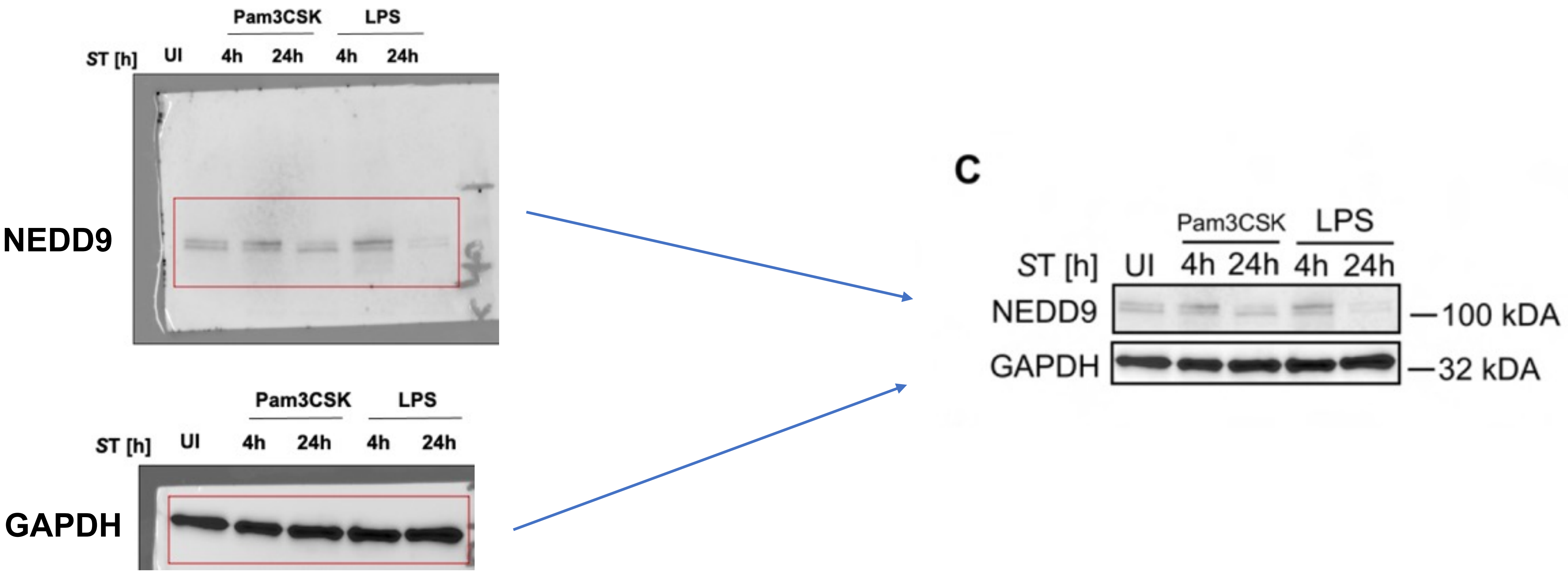

Fig.S1 D

NEDD9

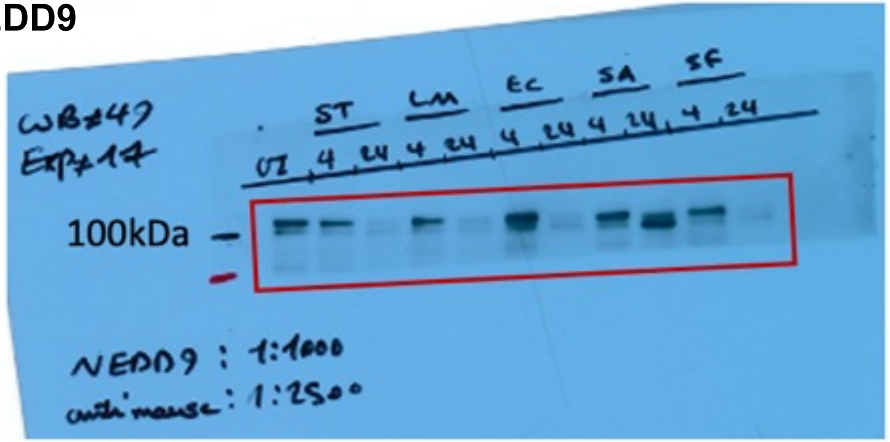

GAPDH

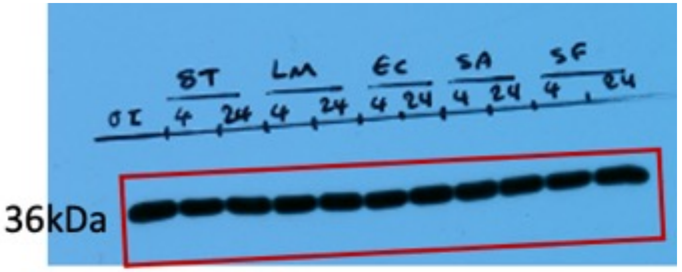

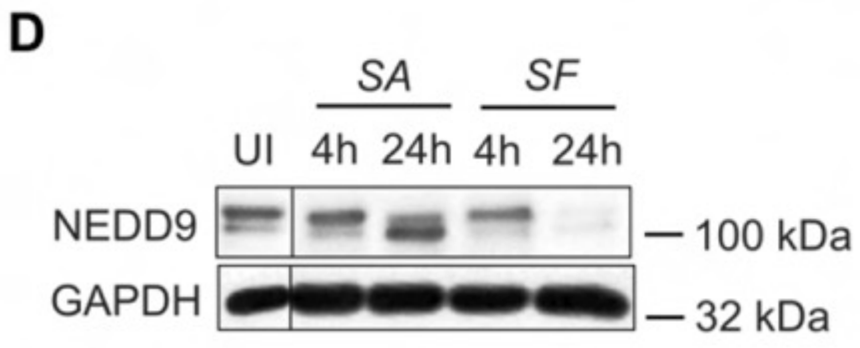

Fig.S2 A

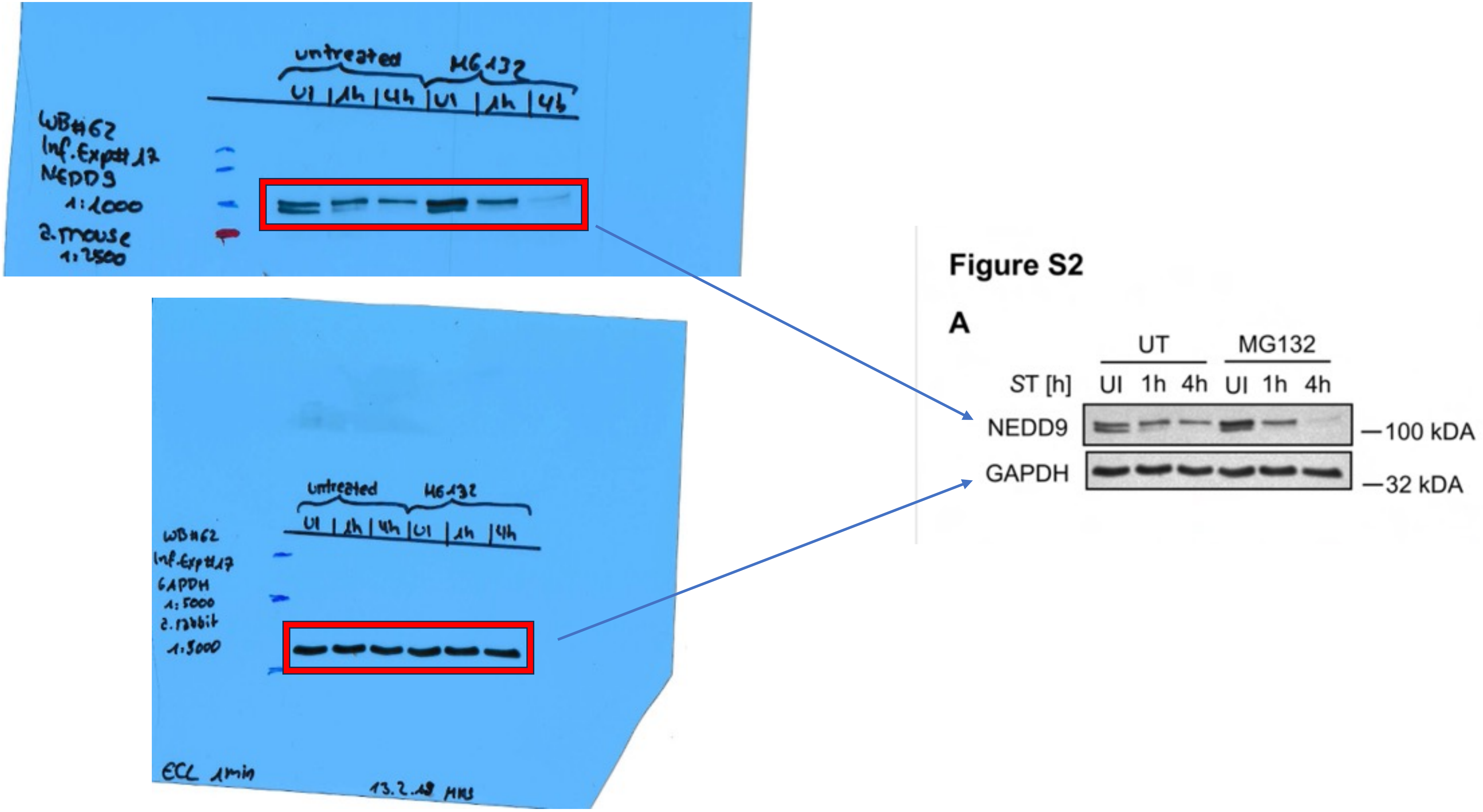
untreated
MG132
UI 1h 4h UI 1h 4h
WB#62
Inf.Expt#17
NEDD9
1:1000
2.mouse
1:2500
GAPDH
1:5000
2.rabbit
1:5000
ECL 1min
Figure S2
A
UT
MG132
ST [h]
UI 1h 4h UI 1h 4h
NEDD9
GAPDH
100 kDA
32 kDA

Fig.S4 A

Fehlen beta-Actin
→ Marc gefragt

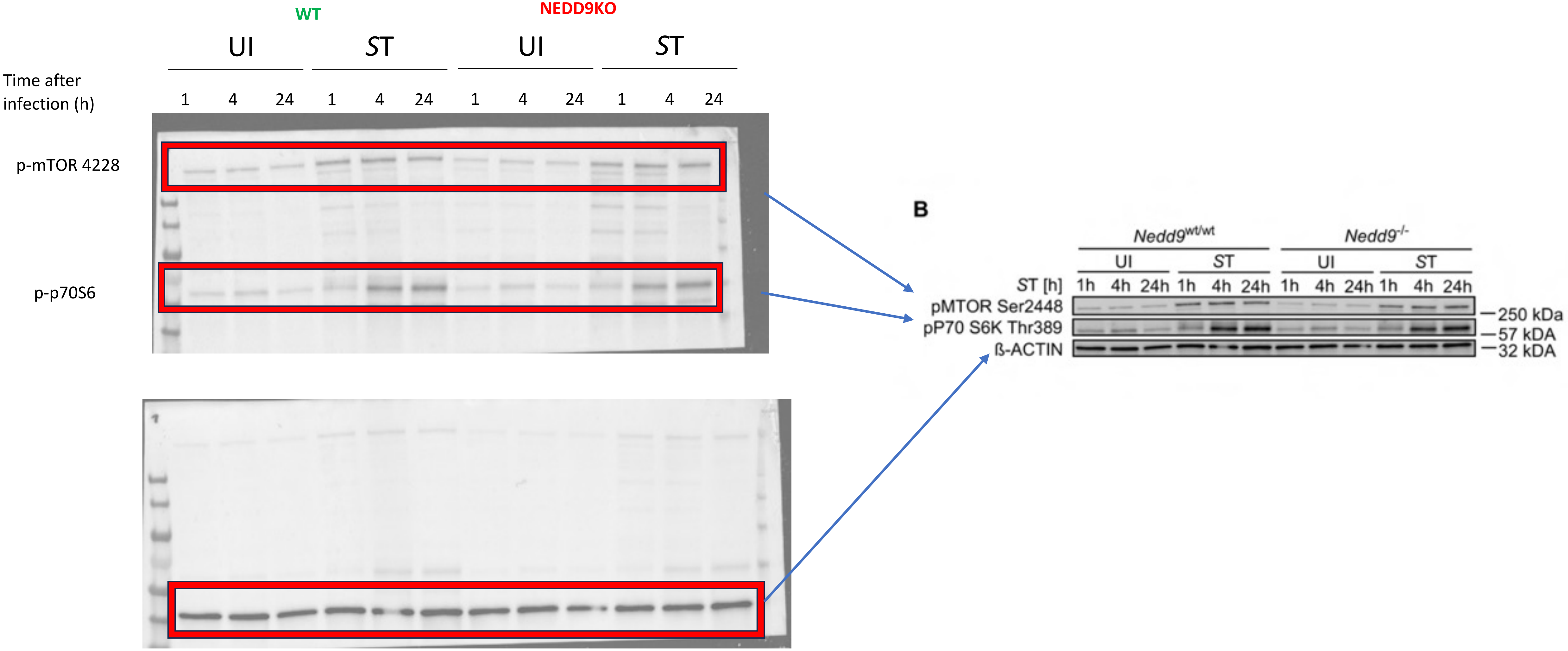

Fig.S4 D

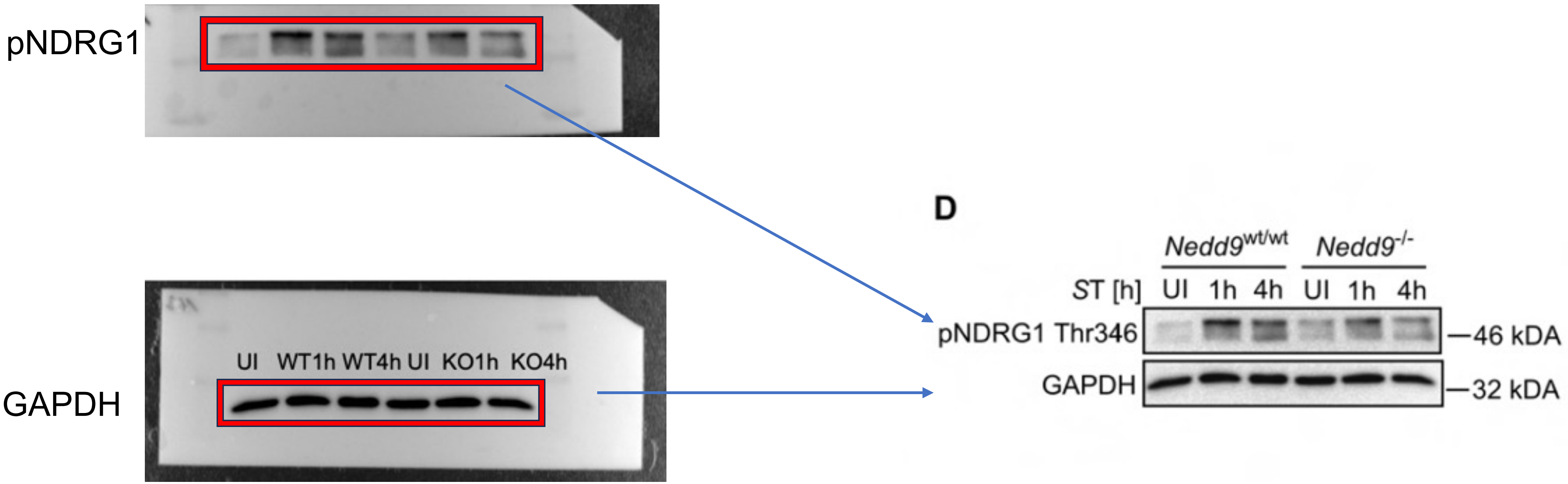
pNDRG1
GAPDH
UI WT1h WT4h UI KO1h KO4h
D
Nedd9wt/wt
Nedd9-/-
ST [h] UI 1h 4h UI 1h 4h
pNDRG1 Thr346
GAPDH
46 kDA
32 kDA

Fig.S4 A

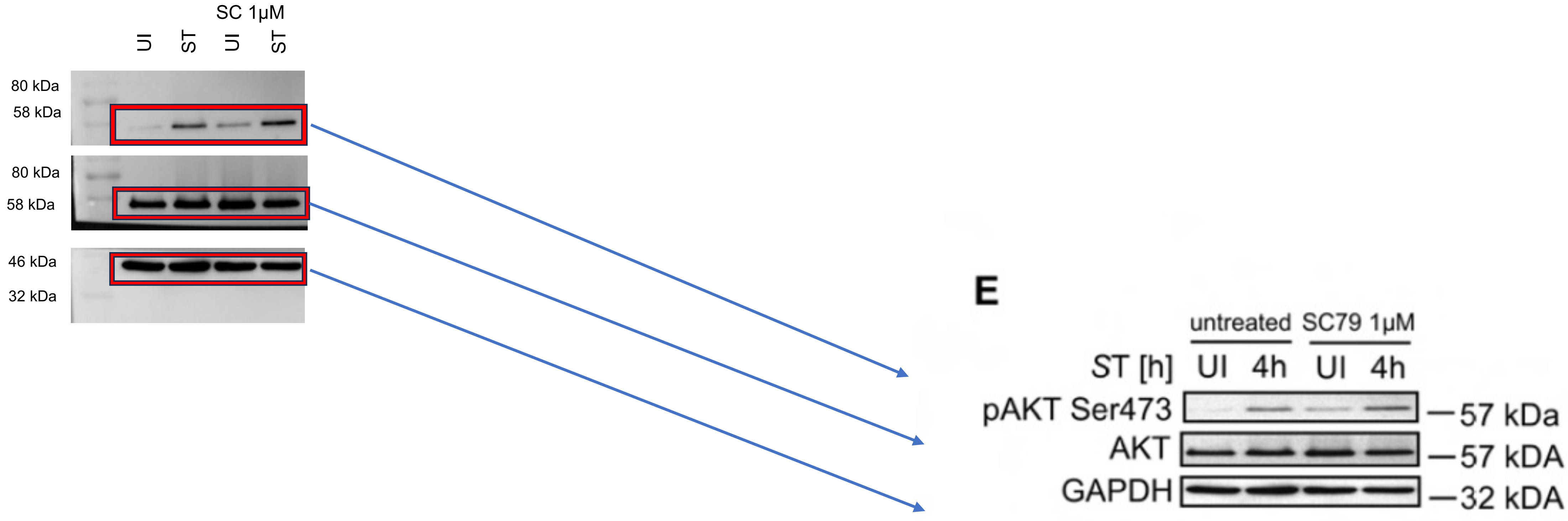
SC 1μM
UI
ST
UI
ST
80 kDa
58 kDa
80 kDa
58 kDa
46 kDa
32 kDa
E
untreated
SC79 1μM
ST [h]
UI
4h
UI
4h
pAKT Ser473
AKT
GAPDH
57 kDa
57 kDA
32 kDA

Fig.S4 A

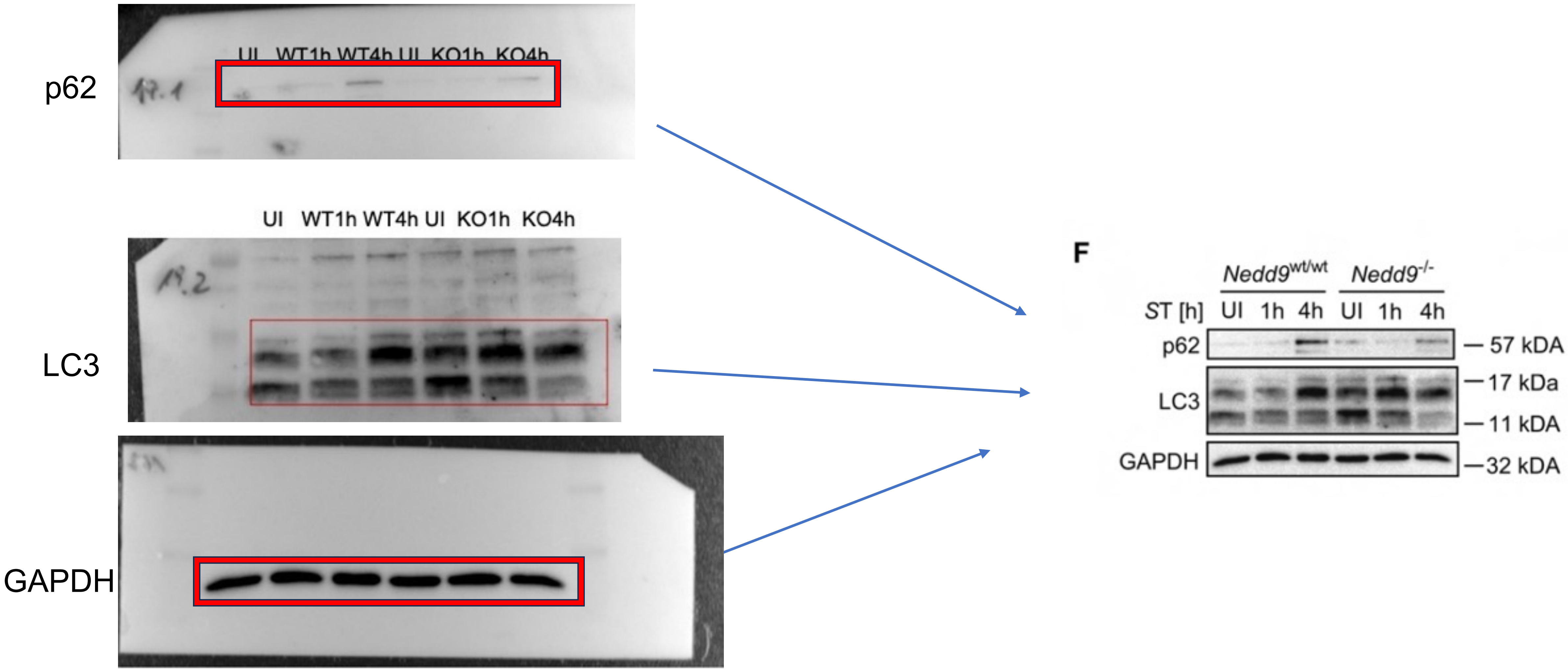

p62
UI WT1h WT4h UI KO1h KO4h
LC3
UI WT1h WT4h UI KO1h KO4h
GAPDH
F
Nedd9wt/wt
Nedd9-/-
ST [h] UI 1h 4h UI 1h 4h
p62
LC3
GAPDH
57 kDA
17 kDa
11 kDA
32 kDA

Fig.1C

UI	ST 4h
0,057502656	0,029610596
5,238050732	0,851189506
1,761274102	0,016503147
11,17758435	0,35880872
10,490964	0,80033233
7,44843466	0,93647771
0,789481	
1,31748832	
2,14140077	
